# Anti-Fungal Drug Anidulafungin Inhibits SARS-CoV-2 Spike-Induced Syncytia Formation by Targeting ACE2-Spike Protein Interaction

**DOI:** 10.3389/fgene.2022.866474

**Published:** 2022-03-25

**Authors:** Shahzaib Ahamad, Hashim Ali, Ilaria Secco, Mauro Giacca, Dinesh Gupta

**Affiliations:** ^1^ Translational Bioinformatics Group, International Centre for Genetic Engineering and Biotechnology, New Delhi, India; ^2^ School of Cardiovascular Medicine and Sciences, British Heart Foundation Centre of Research Excellence, King’s College London, London, United Kingdom; ^3^ Division of Virology, Department of Pathology, Addenbrooke’s Hospital, University of Cambridge, Cambridge, United Kingdom; ^4^ Department of Medical, Surgical and Health Sciences, University of Trieste, Trieste, Italy; ^5^ International Centre for Genetic Engineering and Biotechnology (ICGEB), Trieste, Italy

**Keywords:** SARS-CoV-2, COVID-19, ACE2, virtual screening, MD simulations, syncytia, anidulafungin

## Abstract

Drug repositioning continues to be the most effective, practicable possibility to treat COVID-19 patients. The severe acute respiratory syndrome coronavirus 2 (SARS-CoV-2) virus enters target cells by binding to the ACE2 receptor *via* its spike (S) glycoprotein. We used molecular docking-based virtual screening approaches to categorize potential antagonists, halting ACE2-spike interactions by utilizing 450 FDA-approved chemical compounds. Three drug candidates (i.e., anidulafungin, lopinavir, and indinavir) were selected, which show high binding affinity toward the ACE2 receptor. The conformational stability of selected docked complexes was analyzed through molecular dynamics (MD) simulations. The MD simulation trajectories were assessed and monitored for ACE2 deviation, residue fluctuation, the radius of gyration, solvent accessible surface area, and free energy landscapes. The inhibitory activities of the selected compounds were eventually tested *in-vitro* using Vero and HEK-ACE2 cells. Interestingly, besides inhibiting SARS-CoV-2 S glycoprotein induced syncytia formation, anidulafungin and lopinavir also blocked S-pseudotyped particle entry into target cells. Altogether, anidulafungin and lopinavir are ranked the most effective among all the tested drugs against ACE2 receptor-S glycoprotein interaction. Based on these findings, we propose that anidulafungin is a novel potential drug targeting ACE2, which warrants further investigation for COVID-19 treatment.

## Introduction

COVID-19 consists of a spectrum of syndromes from a mild, flu-like illness to severe pneumonia caused by the severe acute respiratory syndrome coronavirus 2 (SARS-CoV-2) virus (Zhou et al., 2020). Its severity is linked to lung epithelial destruction, thrombosis, and hyperimmune-mediated damage (Bussani et al., 2020; Zhou et al., 2020; Zhu et al., 2020; Buchrieser et al., 2021). Additionally, an abnormal dysmorphic cellular characteristic is the presence of large infected multinucleated cells, predominately comprised of pneumocytes (Bussani et al., 2020; Braga et al., 2021; Sanders et al., 2021). The disease has rapidly spread globally, prompting the WHO in March 2020 to declare it a worldwide pandemic. As per WHO, till February 19, 2022, SARS-CoV-2 is estimated to have infected over 418,650,474 people and caused over 5,856,224 deaths (WHO, 2022).

SARS-CoV-2 is an enveloped virus with a positive-sense single-stranded RNA that belongs to the beta-coronavirus genera of coronaviruses and exploits the human ACE2 receptor to enter the host cells (Hoffmann et al., 2020; Yan et al., 2020), such as SARS-CoV (Wrapp et al., 2020). The spike (S) protein present in the outer envelope of the virus binds the ACE2 receptor expressed on target cells along with other membrane proteins NRP1 (Cantuti-Castelvetri et al., 2020), TMPRSS2 (Hoffmann et al., 2020) and Furin (Johnson et al., 2020; Peacock et al., 2021) (which assist the binding or entry), leading to the access of the virus to the target cells. After binding with the ACE2 molecules, the conformational changes in the S protein lead to the fusion of the viral envelope with the host cell membrane and the subsequent transfer of the RNA viral genome into the cells (Hoffmann et al., 2020). Apart from interacting with ACE2, S protein is also predicted to interact with Glucose Regulated Protein 78 (GRP78) or Bip, which plays a role in virus internalization (Ibrahim et al., 2020; Elfiky and Ibrahim, 2022). Another study reported that the GRP78 is vital for ACE2 trafficking and stability (Carlos et al., 2021). Several groups have conducted computational studies as well as experiments to study the interactions involving S protein and the ACE2 receptor. To target and disrupt the interactions by exploring repurposed drugs or novel inhibitors, attempts have been made to design ligands targeting S protein (Xiu et al., 2020; Wang et al., 2021) as well as ACE2 receptors (Ahmad et al., 2021). Despite the availability of several vaccines to treat COVID-19 and reduce the viral spread and disease severity, COVID-19 still requires novel therapeutics to fight the newly emerging SARS-CoV-2 variants and overcome the significant limitations in vaccine production and distribution, which hamper worldwide effective immunization. Since its appearance, the inherited Wuhan strain has been replaced by variants harboring various mutations in the viral genome (Otto et al., 2021; Singh et al., 2021). Several of these mutations occur in the highly antigenic S protein, which endows several of the variants with the ability to escape part of the neutralizing antibody response (Weisblum et al., 2020; Liu et al., 2021; Planas et al., 2021; Rees-Spear et al., 2021; Starr et al., 2021). Several available vaccines have significantly reduced efficacy against these newly emerged variants (Pouwels et al., 2021; Sanderson, 2021). Various drugs have been proven effective against COVID-19 in controlled clinical trials, including remdesivir (Beigel et al., 2020), corticosteroids (Sterne et al., 2020), and a few monoclonal antibodies (Marovich et al., 2020; Taylor et al., 2021). However, none of these drugs are curative and, in several instances, their clinical effect is quite modest. In addition, some of the available treatments, particularly those with monoclonal antibodies, show diminished activity with the emerging variants. Given the importance of SARS-CoV-2, its transmission and rapid worldwide spread, it is thus crucial to rapidly generate new therapeutic approaches, especially to deal with newly emerging SARS-CoV-2 mutants.

The ACE2 receptor plays an essential role in transmitting the virus to the target host cells. Hence, here we aimed to identify a potential antagonist against the ACE2 receptor, which can inhibit the entry of the virus into human cells. We screened 450 FDA-approved compounds with antiviral properties toward the active pocket of ACE2 receptor using molecular docking-based virtual screening, followed by MD simulation, and subsequently, *in vivo* validation of chosen drugs. Furthermore, MD simulations examined the stability of ligand-protein complexes, and the free energy of binding was calculated using the (MMGB/SA) ΔG methods. Here, we found that two drugs, anidulafungin and lopinavir, effectively block S-induced cell–cell fusion events and S-viral particle entry. As both S-mediated syncytia formation and entry of S-viral particles into the cells require functional ACE2-S protein interactions, we conclude that anidulafungin and lopinavir effectively block the formation of the ACE2-S complex.

## Materials and Methods

### Selection of FDA-Approved Antiviral and ACE2 Structure

Drug candidates were selected among antiviral datasets from published literature to identify the novel drugs which potentially interfere with the SARS-CoV-2 replication by inhibiting spike-ACE2 interactions (Ghahremanpour et al., 2020; Heiser et al., 2020; Jeon et al., 2020; Jin et al., 2020; Ku et al., 2020; Nguyenla et al., 2020; Riva et al., 2020; Touret et al., 2020; Weston et al., 2020; Yuan et al., 2020; Ahamad et al., 2021b; Chen et al., 2021; Dittmar et al., 2021; Ellinger et al., 2021; Ginex et al., 2021; Han et al., 2021; Mirabelli et al., 2021). The structure of the ACE2 receptor (PDB ID: 6M17) was downloaded from the Protein Data Bank (Goodsell et al., 2020).

### Computational Resources

The MD simulations were carried out on High Performance Computing (HPC) cluster of International Business Machines (IBM) Power9 CPU nodes (total 160 CPUs) with NVIDIA TESLA v100 32GB GPUs and Red Hat Enterprise Linux operating system.

### Molecular Docking

To predict the preferred binding pocket on the ACE2 surface, molecular docking-based virtual screening was performed using Flare 5.0 and binding affinities calculated. Flare incorporates BioMolTech’s Lead Finder docking algorithm and combines its docking engine with genetic algorithm search containing local optimization procedures, enabling efficient sampling of ligand poses for refinement. The volume of the grid box was 287,154 A^3^, and the axis was set to be X: 116.403, Y: 97.474, and Z: 183.867 to cover all the amino acids in the box. It includes three different scoring functions (viz., LF dG, LF VSscore, and LF RankScore) for accurately predicting 3D docked ligand poses. The LF RankScore was selected for protein-ligand binding energy and rank ordering of active and inactive compounds in virtual screening experiments. The 2D plot was generated to study residue-ligand interactions, using the Schrödinger Maestro version 12.8.117, release 2021-2 suite (Schrödinger LLC, Cambridge, MA).

### Groningen Machine for Chemical Simulations

We used the general methodology to perform MD simulations of native ACE2 and the best-docked complexes using GROMACS (V5.18.3) (Abraham et al., 2015). For the MD simulation of the docked complexes, suitable force field parameters are required for the ligand/drug topology, which cannot be assigned using GROMACS. Hence, the PRODRG server was used to generate drug topologies and coordinate files (Schuttelkopf and van Aalten, 2004). We used the GROMOS9643a1 force field for native and drug-protein complexes, viz., ACE2-anidulafungin, ACE2-lopinavir, ACE2-indinavir, and ACE2-MLN-4670 (Van Der Spoel et al., 2005). Furthermore, systems were solvated using a Simple Point Charge (SPC) water model in a cubic box (Price and Brooks, 2004). 0.15 M counter ions of sodium (Na^+^) and chlorine (Cl^−^) were added to the simulation box for the system neutralization. All the neutralized systems were energy minimized using the steepest descent followed by conjugate gradient methods (50,000 steps for each). The system equilibration was achieved under the regulation of volume (NVT) and pressure (NPT) ensembles. The NVT ensemble was subjected to a constant temperature of 300 K and a constant pressure of 1 bar. The hydrogen (H) atoms were confined to equilibrium distances and periodic boundary conditions using the SHAKE algorithm. Additionally, the long-range electrostatic forces were defined using the Particle Mesh Ewald (PME) method (Lee et al., 2016). The cut-offs for Van der Waals and Coulombic interactions were set at 1.0 nm (Wang et al., 2016). The bonds and angles were constrained using the LINCS algorithm. Moreover, after a successful NPT ensemble run, the production run was performed for 100 ns. The energy, velocity, and trajectory were updated at a time interval of 10 ps. For the native ACE2 and the complexes, the MD trajectories were analyzed using GROMACS to calculate several parameters, namely, Cα-atom root mean square deviations (RMSD), root mean square fluctuations (RMSF) to investigate the relative fluctuations of each residue, radius of gyrations (Rg) to assess the protein compactness, solvent accessible surface area (SASA) to estimate the electrostatic contributions of molecular solvation, and free energy landscapes (FEL), as described in our previous publications (Ahamad et al., 2021a; Ahamad et al., 2021b).

### Cells

HEK293T cells (ATCC CRL-3216) were cultured in Dulbecco’s modified Eagle medium (DMEM) with 1 g/L glucose (Life Technologies) supplemented with 10% fetal bovine serum (FBS) (Life Technologies) plus a final concentration of 100 IU/ml penicillin and 100 (μg/ml) streptomycin or without antibiotics were required for transfections.

Vero (WHO) Clone 118 cells (ECACC 88020401) were cultured in Dulbecco’s modified Eagle medium (DMEM, Life Technologies) with 1 g/L glucose (Life Technologies) supplemented with 10% heat-inactivated fetal bovine serum (FBS, Life Technologies) plus a final concentration of 100 IU/ml penicillin and 100 (μg/ml) streptomycin or without antibiotics where required for transfection. Cells were incubated at 37°C, 5% CO_2_.

### Plasmids

Human ACE2 (Addgene #1786), pLVTHM/GFP (Addgene #12247), psPAX2 (Addgene 12,260), pMD2.G (Addgene #12259) were obtained from Addgene. pAAV-CMV-GFP was obtained from L. Zentilin (Molecular Medicine Lab, ICGEB). pAAV-spike-V5 and pAA-spike-d19-V5 SARS-CoV-2 spike expression vectors were used previously (Braga et al., 2021).

### Antibodies

Antibodies against the following proteins were used: ACE2 (Abcam, ab15348), SARS-CoV-2 spike (GeneTex GTX632604), V5-488 (Thermo Fisher Scientific, 377500A488), α-beta-actin-HRP (Sigma-Aldrich), mouse-HRP (Abcam, ab6789), and rabbit-HRP (Abcam, ab205718).

### Plasmids DNA Transfections

Plasmid expressing human ACE2 reverse transfection was performed in a 96-well plate; 100 ng of plasmids were diluted in 25µl of Opti-MEM (Life Technologies) and mixed with the transfection reagent (FuGENE HD, Promega) using a ratio of 1 µg pDNA:3 µL FugeneHD. The transfection mixes were incubated for 25 min at RT and added to the 96 well plates (Cell Carrier Ultra 96, Perkin Elmer).

Vero cells (6.5 × 10^3^) or HEK293-ACE2 (8 × 10^3^) cells were seeded in each well. After 24 h of transfections, 100 ng of the pEC117-spike-V5 expression plasmid was transfected using a standard forward transfection protocol. After 24 h, cells were fixed in 4% PFA and processed for immunofluorescence.

### Immunofluorescence

After fixation in 4% PFA for 10 min at RT, cells were washed two times with 1xPBS and then permeabilized in same volumes of 0.1% Triton X100 (Sigma-Aldrich 1086431000) for 10 min at RT. Cells were then washed two times 1xPBS and blocked with 2% BSA for 1 h at RT. After blocking, the cells were stained according to the type of staining.

After blocking, a diluted primary antibody (1:500 in 1% BSA SARS-CoV-2 spike antibody or V5-488) was added to each well and incubated overnight at 4°. Cells were then washed two times with 1xPBS, and then a secondary antibody was added (45 µL/well, diluted 1:500 in 1% BSA) to each well and incubated for 2 h at RT. Cells were then washed two to three times in 1xPBS. Nuclear staining was performed using Hoechst 33,342 (1:5,000).

### Image Acquisition and Analysis

Image acquisition was performed using the Operetta CLS high content screening microscope (Perkin Elmer) with a Zeiss 20× (NA = 0.80) objective, a total of 25 fields were acquired per wavelength, well and replicate (∼10,000–15,000 cells per well and replicate).

Images were subsequently analyzed using the Harmony software (PerkinElmer). Images were first flat field corrected and nuclei were segmented using the “Find Nuclei” analysis module (Harmony). The thresholds for image segmentation were adjusted according to the signal-to-background ratio. The splitting coefficient was set to avoid splitting of overlapping nuclei (fused cells). The intensity of the green fluorescence (spike/GFP) was calculated using the “Calculate Intensity Properties” module (Harmony). All the cells that scored a nuclear area greater than four times (for manual quantification of syncytia, if fused nuclei >3, it counts as a syncytia) the average area of a single nucleus and were simultaneously positive for green (spike) signal in the cytoplasm area were considered as fused or syncytia. Data were expressed as a percentage of fused cells by calculating the average number of fused cells normalized to the total number of cells per well.

For pseudotyped particle entry assays: mean intensities of the segmented nucleus in the 488 (green) channel and the Hoechst channel for each nucleus across all fields were extracted. Each assay plate included a negative control, DMSO. Briefly, nuclei were segmented based on Hoechst staining, and cells were then classified as positive or negative depending on the GFP signal. Data were expressed as a percentage of GFP + cells by calculating the average number of GFP + cells normalized on the total number of cells.

### Pseudotyped Particle Production and Entry Assay

A HIV-1 based lentiviral system was used to produce SARS-CoV-2 spike pseudotyped particles in HEK293T cells by the co-transfection of pMD2.G or pAAV-spike (d19), psPAX2 (packing vector), and PLVTHM (GFP) as described previously (Ali et al., 2019). Viral supernatants were collected after 48 h of transfection and centrifuged at 3,000 rpm for 10 min at 4°. The supernatant was then filtered with a 0.45 µm pore size filter, aliquoted, and stored at −80°C. For pseudotyped particle entry, 1 h before spike pseudotyped particle transduction, HEK293-ACE2 cells were treated with selected drugs and control cells were treated with DMSO. After 36 h, cells were fixed; nuclei were labeled with Hoechst and assessed for pseudotyped particles transduction efficiency based on GFP positive cells.

### Western Blotting

After 20–24 h of drug treatment, Vero cells were processed for western blot analysis. Equal amounts of total cellular proteins (15 μg), as measured with the BCA (Thermofisher, 23,227), were resolved by electrophoresis in 4–20% gradient polyacrylamide gels (Mini-PROTEAN, Biorad) and transferred to nitrocellulose/PVDF membranes (GE Healthcare). Membranes were blocked at RT for 60 min with PBST (PBS + 0.1% Tween-20) and 5% skim milk powder (Cell signalling, 9.999). Blots were then incubated (4°C, overnight) with primary antibodies against ACE2 (diluted 1:1,000), and *α*-tubulin (diluted 1:10,000). Blots were washed three times (10 min each) with PBST. For standard Western blotting detection, blots were incubated with either anti-rabbit HRP-conjugated antibody (1:5,000) or anti-mouse HRP-conjugated antibody (1:10,000) for 1 h at RT. After washing three times at RT with PBST (10 min each), blots were developed with ECL (Amersham).

## Results

### *In Silico* Screening of Inhibitors Targeting ACE2-Spike Protein Interactions

**FIGURE 1:**
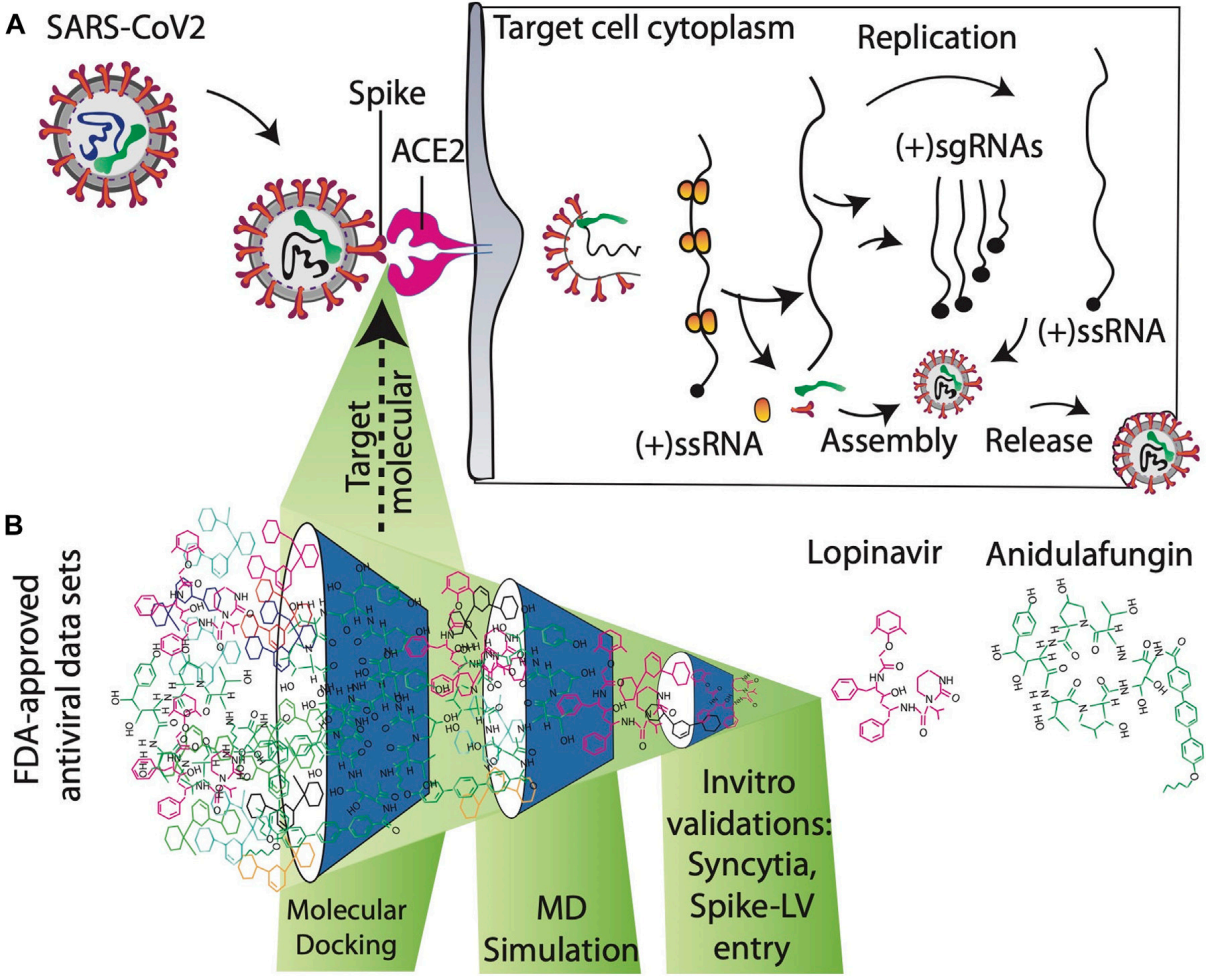
**(A)** Mechanism of action to prevent SARS-CoV-2 entry into the target host cell. SARS-CoV-2 enters human cells after the interaction of the spike protein with the ACE2 receptor. Blocking ACE2-spike interactions by targeting ACE2 receptors with antiviral compounds is an important approach for developing novel therapeutics against SARS-CoV2. **(B)** Schematic overview of searching novel inhibitors at the proposed study.

**FIGURE 2:**
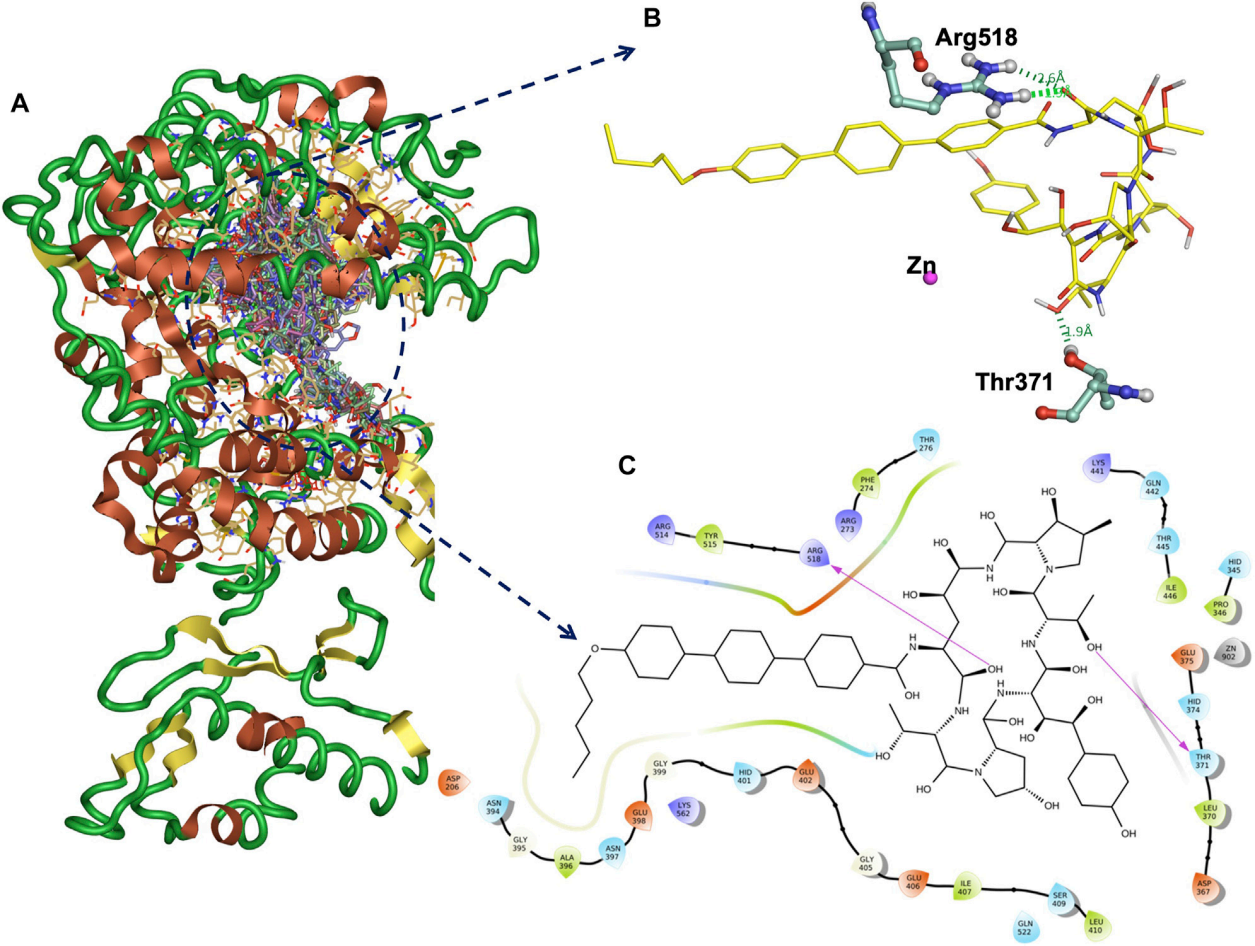
Docked all 450 compounds with ACE2 receptor **(A)**. The binding interaction H-bonds (green) and the amino acid residues of ACE2 and anidulafungin at the binding site **(B)**. The 2D plot of the ACE2-anidulafungin binding pose **(C)**.

Functional ACE2-S interaction is essential for SARS-CoV-2 entry into host cells, as shown in (Figure 1A). Detailed structural analysis proved that both SARS-CoV-2 and SARS-CoV S proteins strongly bind to ACE2 receptors (Lan et al., 2020; Magro et al., 2021). Therefore, to identify drugs that could inhibit the ACE2-S interactions and potentially viral replication, we screened the 450 drugs (Supplementary Table S1) by exploiting the molecular docking approach (Figures 1B, 2A–C). Here, we found that several compounds show a strong affinity toward the ACE2 receptor, but we selected the top three compounds, namely, anidulafungin, lopinavir, and indinavir, showing more binding affinity than MLN-4670, which is a known enzymatic inhibitor of ACE2 (Dales et al., 2002a) (Figures 2B, C, Supplementary Figures S1A–C). Detailed molecular interactions and properties of the four selected docked complexes are shown in Table 1. In the ACE2-anidulafungin complex, anidulafungin forms two hydrogen(H) bonds with Arg518 and Thr371 amino acid residues (Figures 2B, C) and hydrophobic bonds with 32 amino acids (shown in Table 1) together with one Zn-ion at the binding pocket of the ACE2 receptor. Similarly, in the lopinavir-ACE2 complex, lopinavir forms 1-H bonds with Glu398 and hydrophobic bonds with 38 amino acids (shown in Table 1). Indinavir interacts with Gly395 and Glu402 through 2H-bonds, hydrophobic bonds with 32 amino acid residues and Zn ions (shown in Table 1). The docking and 2D plot are shown in (Supplementary Figures S1D–F). However, MLN-4760 interacts with ACE2 by forming one H-bond with Glu402 (Supplementary Figures S1C–F) and hydrophobic bonds with 25 amino acid residues and a Zn metal ion (shown in Table 1).

**TABLE 1:** Physicochemical properties of anidulafungin, lopinavir, indinavir, and MLN-4760.

Compounds	Anidulafungin	Lopinavir	Indinavir	MLN-4670
MW	1140.3	628.8	613.8	428.3
Atoms	82	46	45	28
SlogP	2.1	4.7	3.6	3.4
TPSA	377.4	120	118	104.4
RB	38	16	14	12
dG	−12.96	−8.77	−8.93	−11.11
LF VSscore	−14.24	−11.05	−10.56	−11.46
LF RankScore	−14.42	−12.97	−12.94	−9.78
H-bonds	Arg518 and Thr371	Glu398	Gly395 and Glu402	Glu402
Residues forming hydrophobic interactions	Asp206, Arg273, Phe274, Thr276, Asp367, Leu370, Thr371, His345, Pro346, His374, Glu375, Asn394, Gly395, Ala396, Asn397, Glu398, Gly399, His401, Glu402, Gly405, Glu406, Ile407, Ser409, Leu410, Lys441, Gln442, Thr445, Ile446, Gln522, Arg514, Tyr515, Lys562 and Zn	Phe40, Asp206, Tyr207, Arg273, His345, Pro346, Thr347, Ala348, Trp349, Asp350, Leu351, His374, Glu375, His378, Ile379, Tyr381, Asp382, Tyr385, Arg393, Asn394, Gly395, Ala396, Asn397, Glu398, Gly399, Phe400, His401, Glu402, Ala403, Ile513, Arg514, Tyr515, Tyr516, Thr517, Arg518, Thr519, Tyr521, Lys562 and Zn	Phe40, Asp206, His345, Pro346, Thr347, Ala348, Trp349, Asp350, Leu351, Gly352, Phe356, His374, Glu375, His378, Tyr381, Asp382, Tyr385, Phe390, Arg393, Asn394, Ala396, Asn397, Glu398, Gly399, Phe400, His401, Ala403, Arg514, Tyr515, Thr517, Arg518, Thr519 and Zn	Arg273, His345, Pro346, Thr347, Ala348, Met360, Asp367, Asp368, Thr371, His374, Glu375, His378, Asn397, Glu398, Gly399, Phe400, His401, Ala403, Gly405, Glu406, His505, Arg514, Tyr515, Tyr516, Arg518 and Zn
MMGBSA (ΔG)	−162.28	−67.19	−92.1	−73.53

Out of all the tested compounds, three drugs show high binding affinities toward ACE2 compared to MLN-4760, a known enzymatic inhibitor of ACE2. So, potentially, these drugs might inhibit SARS-CoV-2 replication by interfering with the formation of functional ACE2-S interactions.

### Anidulafungin Forms the Most Stable Complex With ACE2 Receptor in the Molecular Dynamics Simulations

To investigate molecular interactions of the docked complexes further, we performed MD simulations of ACE2-native, four selected docked complexes (ACE2-anidulafungin, ACE2-lopinavir, ACE2-indinavir) and ACE2-MLN-4670 for 100 ns. The stability, interaction profile, and structural parameters including RMSD, RMSF, Rg, SASA, and free energy calculations were also evaluated throughout the simulation run time to select the most stable receptor-drug complex.

**FIGURE 3:**
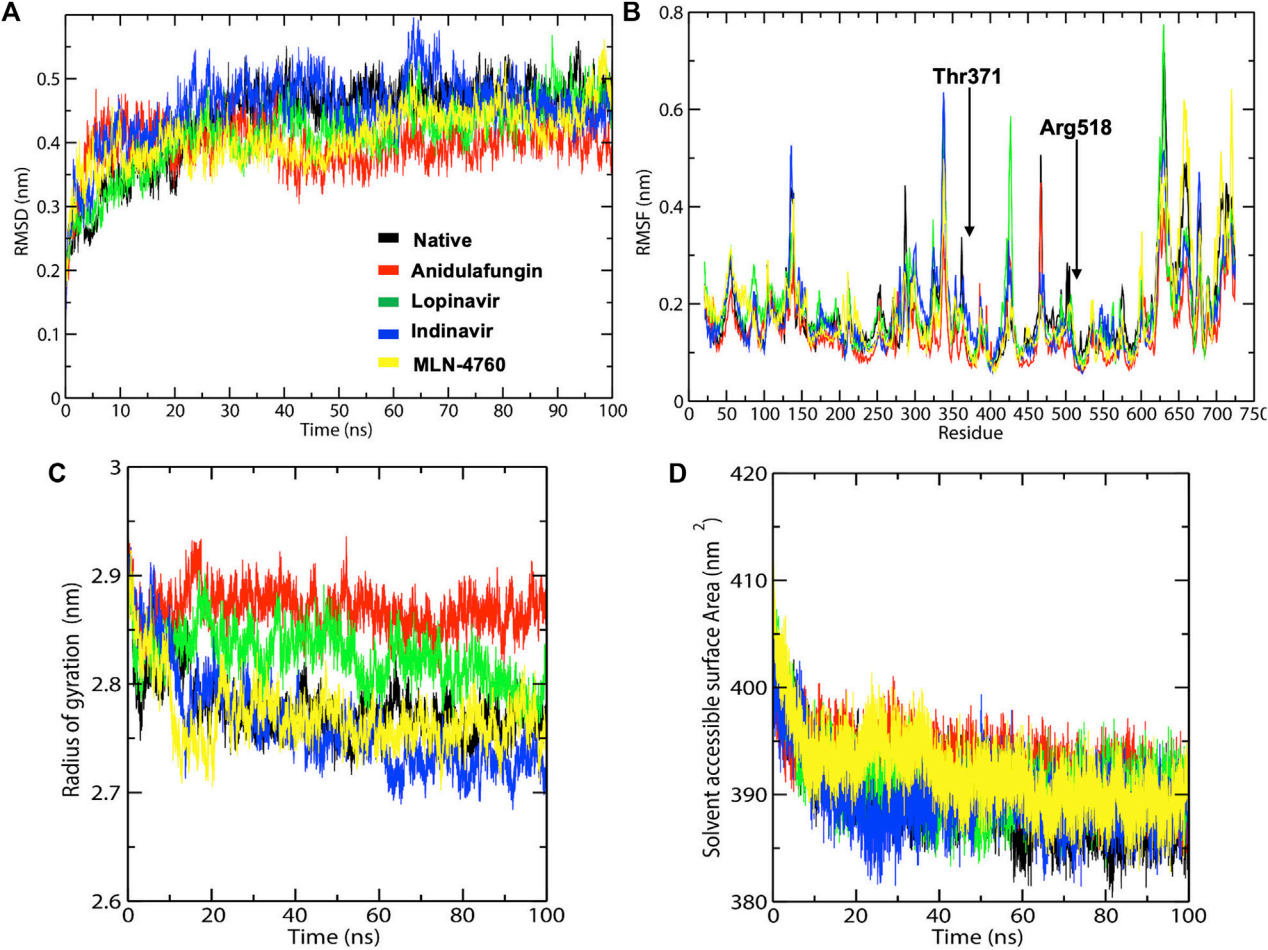
The elucidation of MD simulation of native ACE2 and ACE2-docked complexes. **(A)** Representation of C-alpha conformation of RMSD. **(B)** Comparative RMS fluctuation plot of native ACE2 and ACE2-docked complexes. **(C)** Rg analysis of native ACE2 and ACE2-docked complexes. **(D)** SASA plot of native ACE2 and ACE2-docked complexes.

In RMSD analysis, native ACE2 showed steady RMSD and revealed a threshold of ∼0.44 nm toward the binding with ACE2 under given simulation conditions (Figure 3A). The docking complexes of ACE2 with lopinavir, indinavir, and MLN-4760 noticeably reached equilibrium with average RMSD values of 0.45, 0.41, and 0.41 nm, respectively. The compound lopinavir revealed a high drift in the average RMSD values. However, the average RMSD values of indinavir and MLN-4760 remained the same.

The RMS deviation of Cα-atoms remained stable throughout the simulation with a slight difference in the values but proposed one complex with anidulafungin, indicating strong binding due to polar interaction with Arg518 and Thr371 residues as well as various non-polar interactions. Anidulafungin displayed the least RMSD fluctuations at the ACE2 binding pocket compared to the other drug compounds. The overall results suggested that the anidulafungin was reliably stable among all the complexes.

Secondly, RMS-fluctuations play a crucial role in identifying the flexible and rigid regions of drug-receptor complexes. Hence, RMSF calculations were performed to measure the average atomic flexibility of the ACE2 receptor Cα-atoms alone and in complex with the tested compounds. The average RMSF values were recorded as —Native-ACE2 (0.18 nm), anidulafungin (0.14 nm), lopinavir (0.18 nm), indinavir (0.15 nm) and MLN-4760 (0.18 nm) (Figure 3B). Interestingly, we observed that the ACE2-anidulafungin complex showed a low degree of fluctuations compared to other docked complexes and native-ACE2. However, lopinavir and indinavir displayed the highest degree of fluctuations and hence comparatively less stable. The above-mentioned comparative analysis of Cα-RMSF confirms a high level of flexibility caused by the presence of drug molecules on the protein structure in comparison to the native.

Next, we analyzed the compactness of the native ACE2 and docked complexes by using the radius of gyration (Rg) calculations. The results showed that the Rg values of the native-ACE2 receptor and the complexes of anidulafungin, lopinavir, indinavir, and MLN-4760 compounds remained highly stable with ranges of 2.78, 2.86, 2.82, 2.76, and 2.77 nm, respectively, throughout the MD simulation period (Figure 3C; Table 2 **)**. The low oscillations in Rg and SASA values portrayed high stability for the anidulafungin-ACE2 complex compared to other complexes and ACE2 alone. Interestingly, the Rg results also revealed that the ACE2-anidulafungin complex is the most stable of all the tested complexes. Comparative analysis of the Rg values shows the folding behavior of ACE2 upon binding with anidulafungin, which indicates high compactness between the complexes. We also performed a SASA analysis to better understand the solvent behavior of native ACE2 and docked complexes. Here, we found an average value of native ACE2, anidulafungin, lopinavir, indinavir, and MLN-4760 complexes of 389.88, 392.60, 391.05, 389.90, and 392.10 nm^2^, respectively (Figure 3D; Table 2). These results showed that the compound anidulafungin possessed more stable hydrophobic contacts than the other docked complexes, making most of the ACE2 receptor surface accessible to the solvent and other molecules.

**TABLE 2:** The average values of RMSD, Rg, and SASA of the native ACE2 and complex containing compounds anidulafungin, lopinavir, indinavir, and MLN-4760.

Complexes	Average RMSD (nm)	Average RMSF (nm)	Average SASA (nm^2^)	Average Rg (nm)
Native-ACE2	0.44	0.18	389.88	2.78
ACE2-anidulafungin	0.39	0.14	392.60	2.86
ACE2-lopinavir	0.41	0.18	391.05	2.82
ACE2-indinavir	0.45	0.15	389.90	2.76
ACE2-MLN-4760	0.41	0.18	392.10	2.77

**FIGURE 4:**
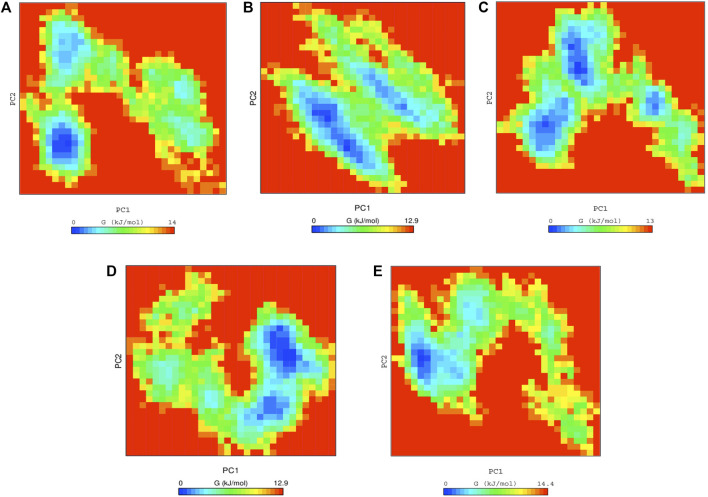
Free energy landscape analysis of **(A)** native ACE2 and complexes with Anidulafungin **(B)**, lopinavir **(C)**, indinavir **(D)**, and MLN-4760 **(E)** compounds.

Finally, the docked complexes were also subjected to the overall motion of all protein and drug atoms by using Free Energy Landscapes (FEL) analysis. The conformational stabilities of the native ACE2 and the docked complexes were examined by FEL analysis using PC1 (Principal Components) and PC2 values. The values of FEL ranged from 0 to 14, 12.9, 13, 12.9, and 14.4 kJ/mol for the native ACE2, anidulafungin, lopinavir, indinavir, and MLN-4760 docked complexes, respectively **(** Figures 4A–E **)**. This analysis indicated that the complexes were stable and persistent energy minima, suggesting the amino acids of the ACE2 binding pocket-forming interactions with drugs are vital for the stability and interaction. The global free energy minima results showed that the docked complexes revealed stabilizing effect that lead to the observed folding behavior of ACE2 with anidulafungin. The analysis revealed that the anidulafungin (Figure 4B) has fewer basins compared to the native receptor with three basins (Figure 4A). Overall, the results for the anidulafungin complex revealed the presence of two basins in the conformational space, with distinct global free energy minima, which consequently lead to a more stable behavior of the protein.

### Anidulafungin and Lopinavir Inhibit SARS-CoV-2 Spike-Induced Syncytia Formation

**FIGURE 5:**
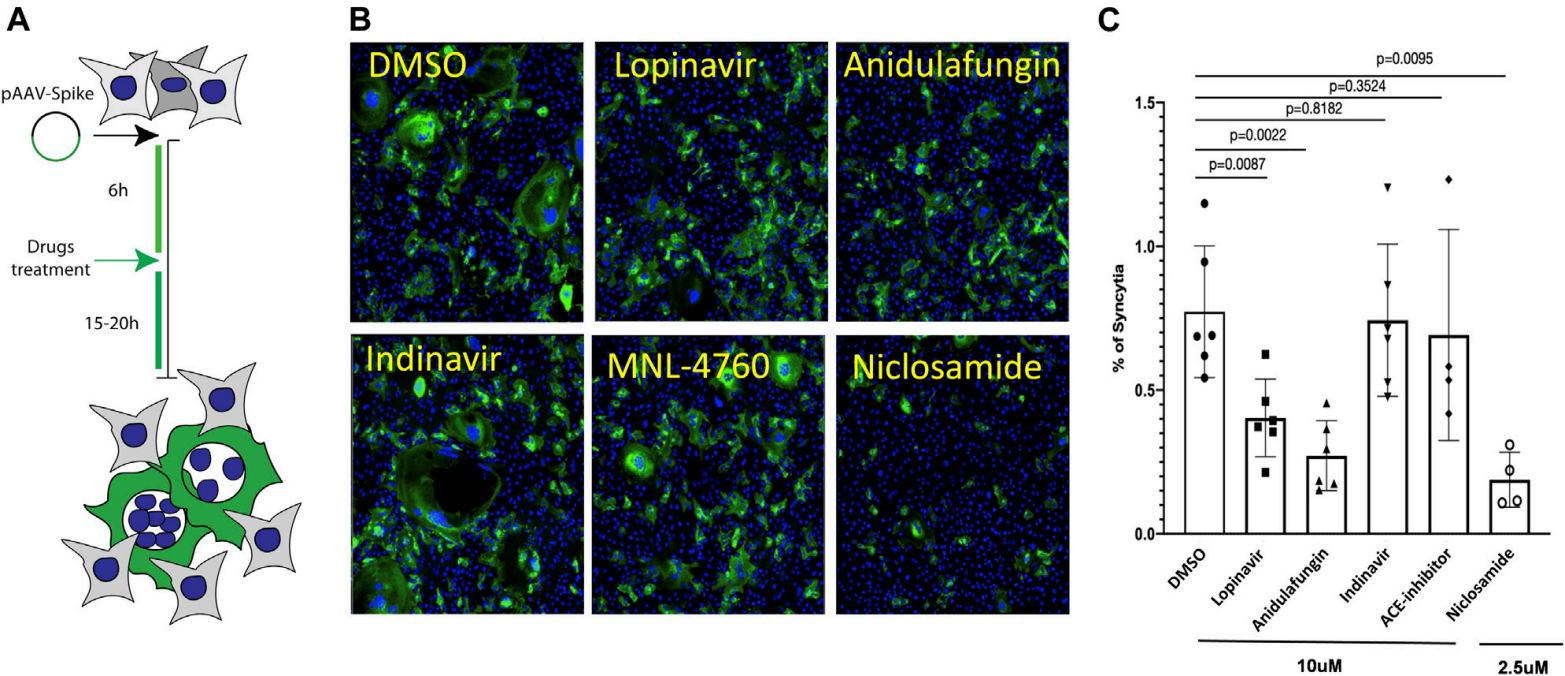
Anidulafungin and lopinavir impaired the spike-mediated syncytia formation. **(A)** Schematic representation of the SARS-Cov2 spike-mediated cell–cell fusion assay. **(B)** Vero cells were treated with either DMSO or top 3 selected drugs after 6 h of spike expressing plasmid transfection. After 20 h, cells were immunostained with anti-spike (green) and nuclei (blue). **(C)** Quantifications. Data (mean ± SD; *n* = 6, Mann-Whitney U test) are plotted as the percentage of fused cells (syncytia) normalized on the total number of cells.

The SARS-CoV-2 spike protein in the viral envelope is essential for virus entry into the target cells. The SARS-CoV-2 S protein induces cell–cell fusion and the formation of syncytia when it is ectopically expressed on the membrane of host cells and binds ACE2 receptors of adjacent cells (Bussani et al., 2020; Xia et al., 2020; Braga et al., 2021; Buchrieser et al., 2021; Gutmann et al., 2021). Therefore, we explored whether S-induced syncytia formation would be impaired in the presence of the selected drugs, such as anidulafungin, lopinavir, indinavir, and MLN-4760, which show high affinity toward the ACE2 receptor in an *in-silico* analysis (Figure 2; Supplementary Figure S1). First, we tested the effect of drugs on S-mediated syncytia formation in Vero cells, which our previous studies have shown to respond to S expression by fusion (Braga et al., 2021). After 6 h of S-protein expression, cells were treated with the indicated drugs at 10 μM (Workflow shown in Figure 5A). Niclosamide (2.5 μM) was used as a positive control because this drug is a potent inhibitor of S-mediated syncytia formation by acting on the cellular TMEM16F membrane protein (Braga et al., 2021). Interestingly, we observed that both anidulafungin and lopinavir treatment significantly reduced the S-mediated cell–cell fusion compared with DMSO-treated control cells (Figures 5B, C). As expected, niclosamide treatment significantly reduced syncytia formation (Figures 5B,C). Both indinavir (Vacca et al., 1994; Condra et al., 1996) and MLN-4760 (Dales et al., 2002b; Joshi et al., 2016) were ineffective in blocking S-mediated cell fusion (Figures 5B, C). None of the tested drug treatments interfered with ACE2 expression in the cells (Supplementary Figure S2). Additionally, we neither observed significant toxicity of the tested drugs (Supplementary Figure S3) nor any effect on S-transgene expression (Supplementary Figure S4). Together, these results are consistent with the conclusion that the observed effects of both anidulafungin and lopinavir are due to interference between ACE2-S interactions.

### Anidulafungin and Lopinavir Inhibit SARS-CoV-2 Spike-Viral Particle Entry

**FIGURE 6:**
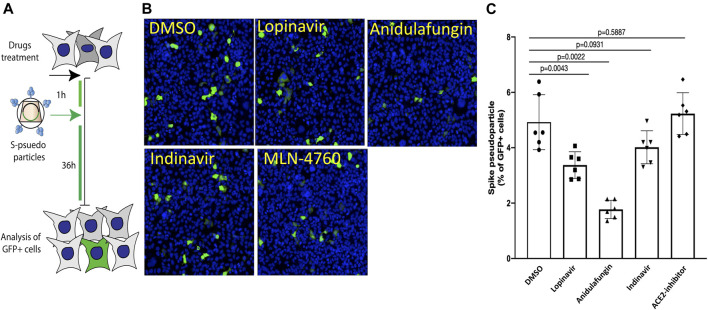
Anidulafungin impaired the spike-pseudotyped particle internalization. **(A)** Schematic representation of the SARS-Cov2 spike pseudotyped particle sentry assay. **(B,C)** HEK293/ACE2 cells were pre-treated with indicated drugs 1–2 h before adding the spike pseudotyped particles carrying GFP as a reporter. After 36 h, cells were immunostained with anti-GFP (green) and nuclei (blue). Representative images are in panel **(B)** (spike pseudotyped particles), and quantifications in panel **(C)** (spike pseudotyped particles). Data (mean ± SD; *n* = 6, *Mann-Whitney U test*) are plotted as the percentage of GFP + cells normalized on the total number of cells.

Entry of SARS-CoV-2 S-viral particles mimics the entry pathway of SARS-CoV-2 virions (Lu et al., 2020; Mykytyn et al., 2021). Therefore, we explored whether the entry of pseudotyped lentiviral vectors expressing S on their envelope would be impaired in the presence of the top selected drugs. For this purpose, HEK cells expressing the ACE2 receptor were treated with the indicated drugs 1 h before the addition of S-pseudotyped viral particles to the cells (Workflow shown in Figure 6A). Strikingly, we found that both anidulafungin and lopinavir treatment significantly impaired S-pseudotyped particle transduction (Figures 6B,C), while no significant effect was observed in the presence of indinavir and MLN-4760. In particular, anidulafungin, which showed the strongest affinity toward the ACE2 receptor in our *in-silico* analysis and formed the most stable complex with ACE2 throughout the MD simulation period, was also the most effective in blocking S-mediated virion internalization.

Collectively, these results indicate that anidulafungin impedes both S-mediated syncytia formation and S-viral particle entry into the target cells.

## Discussion

In this work, we screened the FDA-approved antiviral dataset using the molecular docking approach and selected the best three compounds, namely, anidulafungin, lopinavir, and indinavir, which show strong binding affinity toward the ACE2 receptor.

Both lopinavir and indinavir are antiretroviral drugs that inhibit HIV-1 replication by targeting viral protease (Lv et al., 2015). Growing pieces of evidence suggest that lopinavir has antiviral activity against SARS-CoV-2 (Choy et al., 2020). It has been proposed that it also inhibits the action of the SARS-CoV-2 protease 3CLpro, hence disrupting the viral replication process (Anand et al., 2003; Zhang et al., 2020). However, coronavirus proteases, including 3CLpro, do not contain a C2-symmetric pocket, which is the target of HIV protease inhibitors (Li and De Clercq, 2020; Sheahan et al., 2020). Moreover, darunavir, another HIV protease inhibitor, is ineffective against SARS-CoV-2, as revealed in a non-peer reviewed *in vitro* study (Johnson et al., 2020). Therefore, the reported anti-SARS-CoV-2 effects of lopinavir might be due to its affinity toward the ACE2 receptor, which leads to disruption of ACE2-spike interaction; however, this requires further validation.

Anidulafungin is an anti-fungal lipo-peptide drug approved to treat invasive candidiasis, candidemia, and esophageal candidiasis. It targets the critical enzyme 1,3-β-D-glucan synthase, essential for fungal cell wall synthesis (Debono et al., 1995). In MD simulations, all the three drug complexes with ACE2 were more stable than native-ACE2 and MLN-4670 inhibitors. Moreover, the anidulafungin-ACE2 docked complex was most stable during MD analysis and exhibited an excellent binding affinity and energy of −14.42 kcal/mol and ΔG −162.28 kcal/mol, respectively. The MD simulation analysis also confirmed that the anidulafungin-ACE2 complex is stable, indicating that it can effectively block the ACE2 receptor sites by interacting with critical amino acid residues. Recently, an *in-silico* study has also shown that anidulafungin has an affinity toward ACE2 receptors (Ahsan and Sajib, 2021).

The SARS-CoV-2 S protein plays a significant role in host cell viral attachment to receptor ACE2, and it also induces cell–cell fusion once expressed on the plasma membrane of ACE2-expressing cells. In our experiments, anidulafungin and lopinavir effectively blocked S-induced syncytia formation and S-pseudotyped particle entry into ACE2-expressing, target cells. Of interest, MLN-4760, an enzymatic inhibitor of ACE2 (Dales et al., 2002a), was ineffective in both blocking syncytia formation and S-pseudotyped particle entry. This indicates the relevance of ACE2 in S-mediated cell fusion and strengthens the conclusion that our top-performing drugs are effective by directly acting on this receptor.

Our work discloses two drugs that appear to deserve further consideration as antiviral drugs for COVID-19 patients. However, further studies are required to fully understand their mechanism of action and potency against infectious SARS-CoV-2.

### Statistical Analysis

Mann-Whitney U significance test was used for the data analysis.

## Data Availability

The original contributions presented in the study are included in the article/Supplementary Material; further inquiries can be directed to the corresponding authors.
