# Supplementary figures and images for: Anti-Fungal Drug Anidulafungin Inhibits SARS-CoV-2 Spike-Induced Syncytia Formation by Targeting ACE2-Spike Protein Interaction
Source: Front Genet. 2022 Mar 25;13:866474. doi: 10.3389/fgene.2022.866474 (PMC8990323; doi:10.3389/fgene.2022.866474)

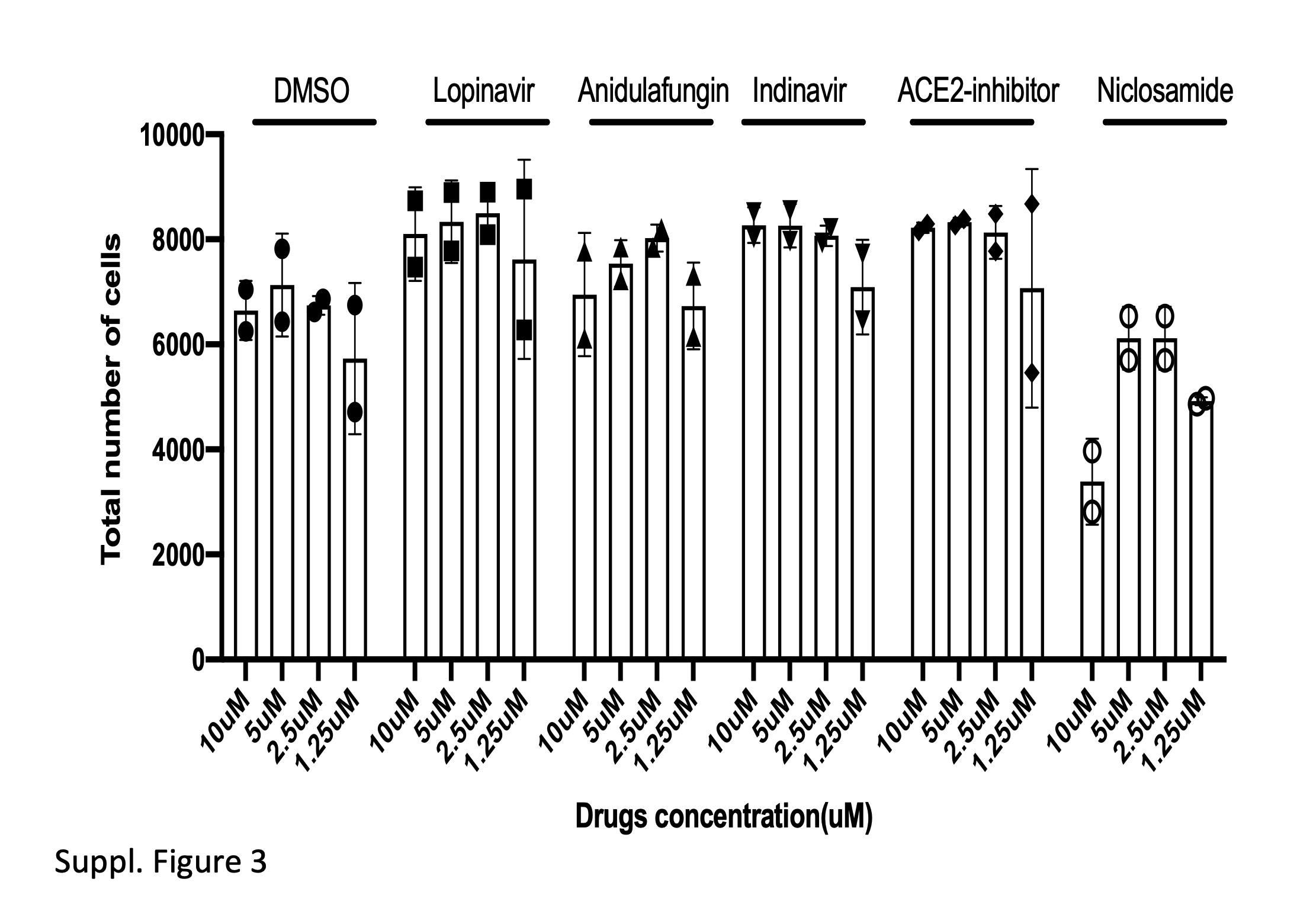

Supplement: Supplementary file 1 [file Image3.JPEG]

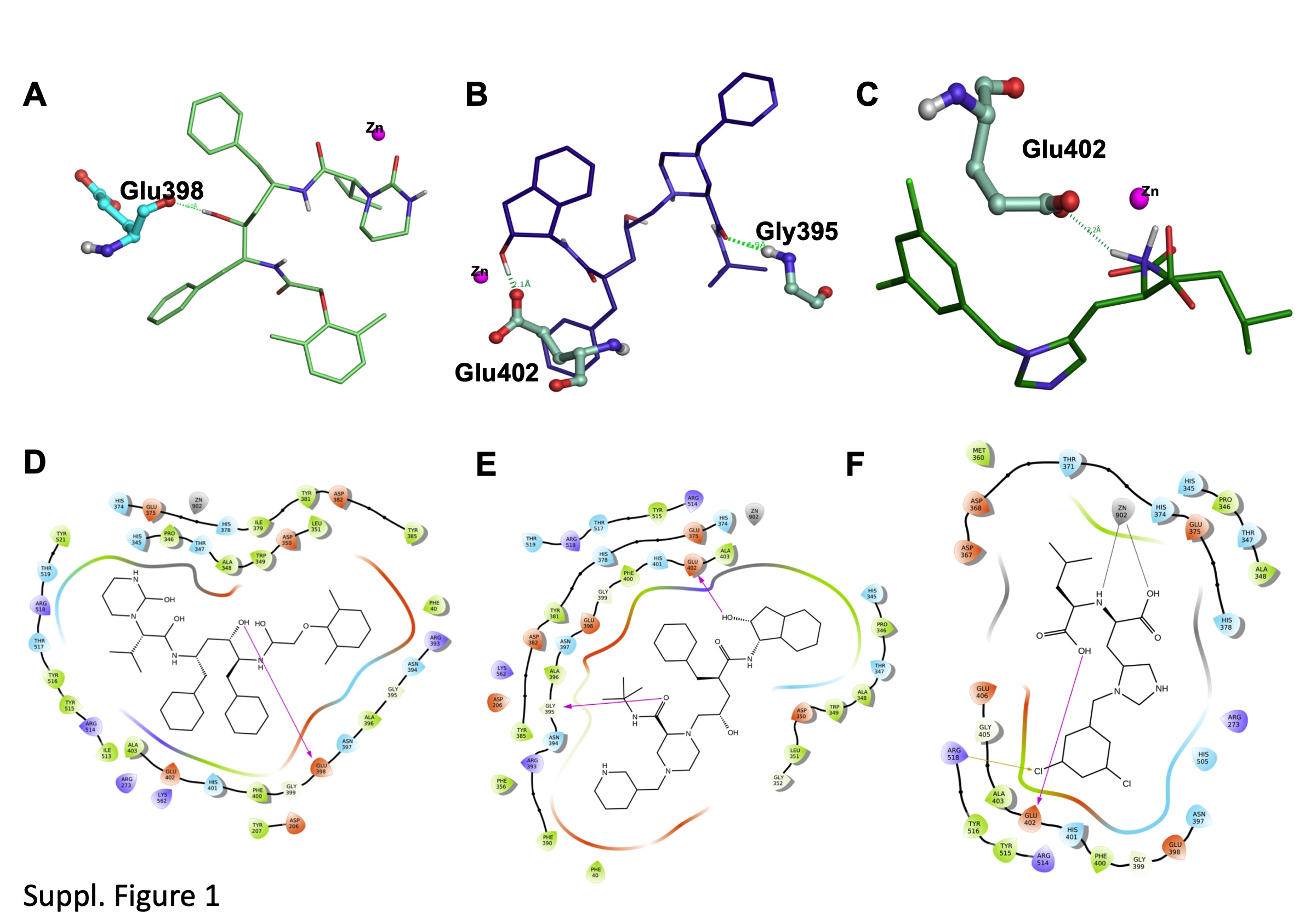

Supplement: Supplementary file 3 [file Image1.JPEG]

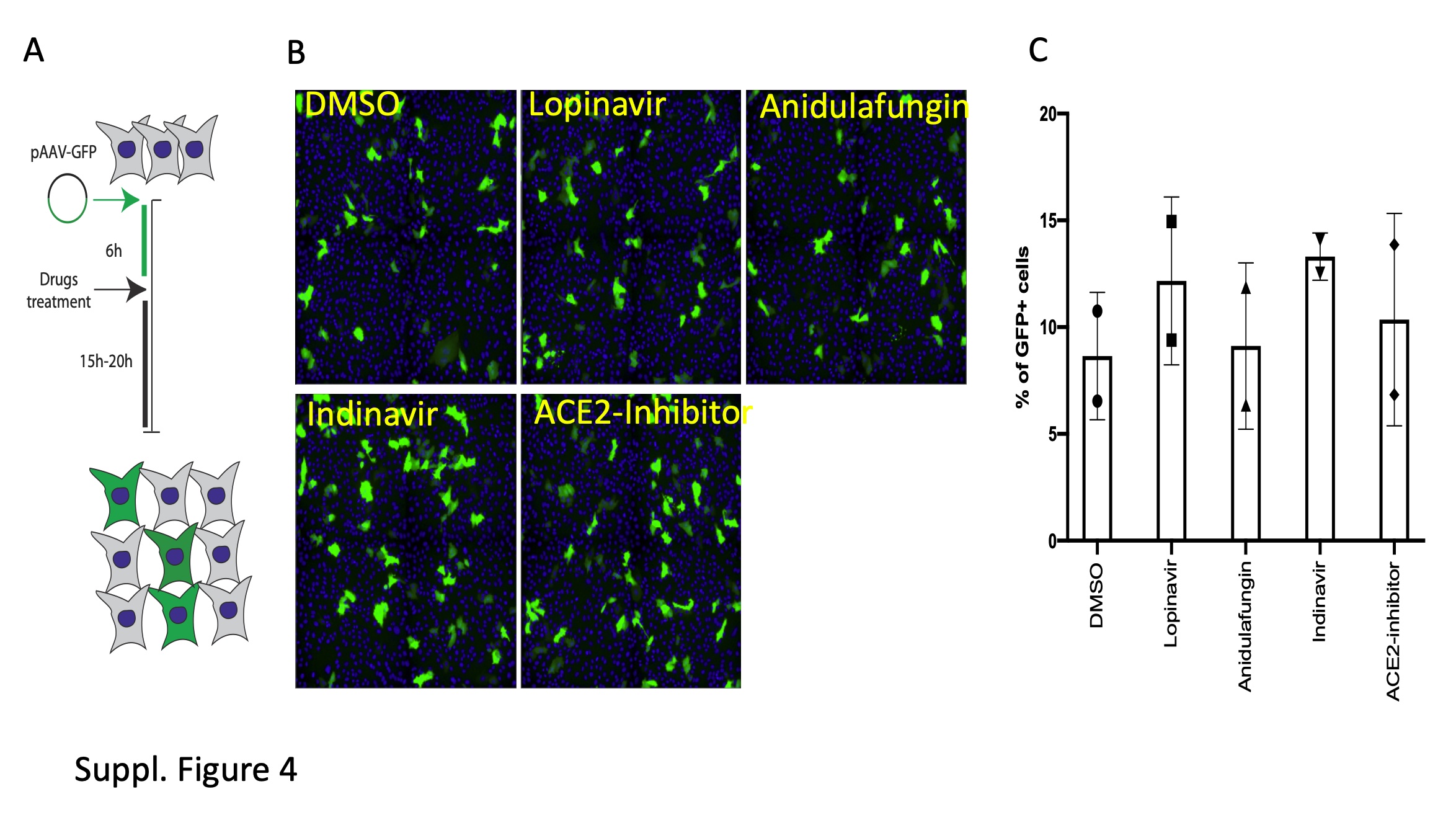

Supplement: Supplementary file 4 [file Image4.JPEG]

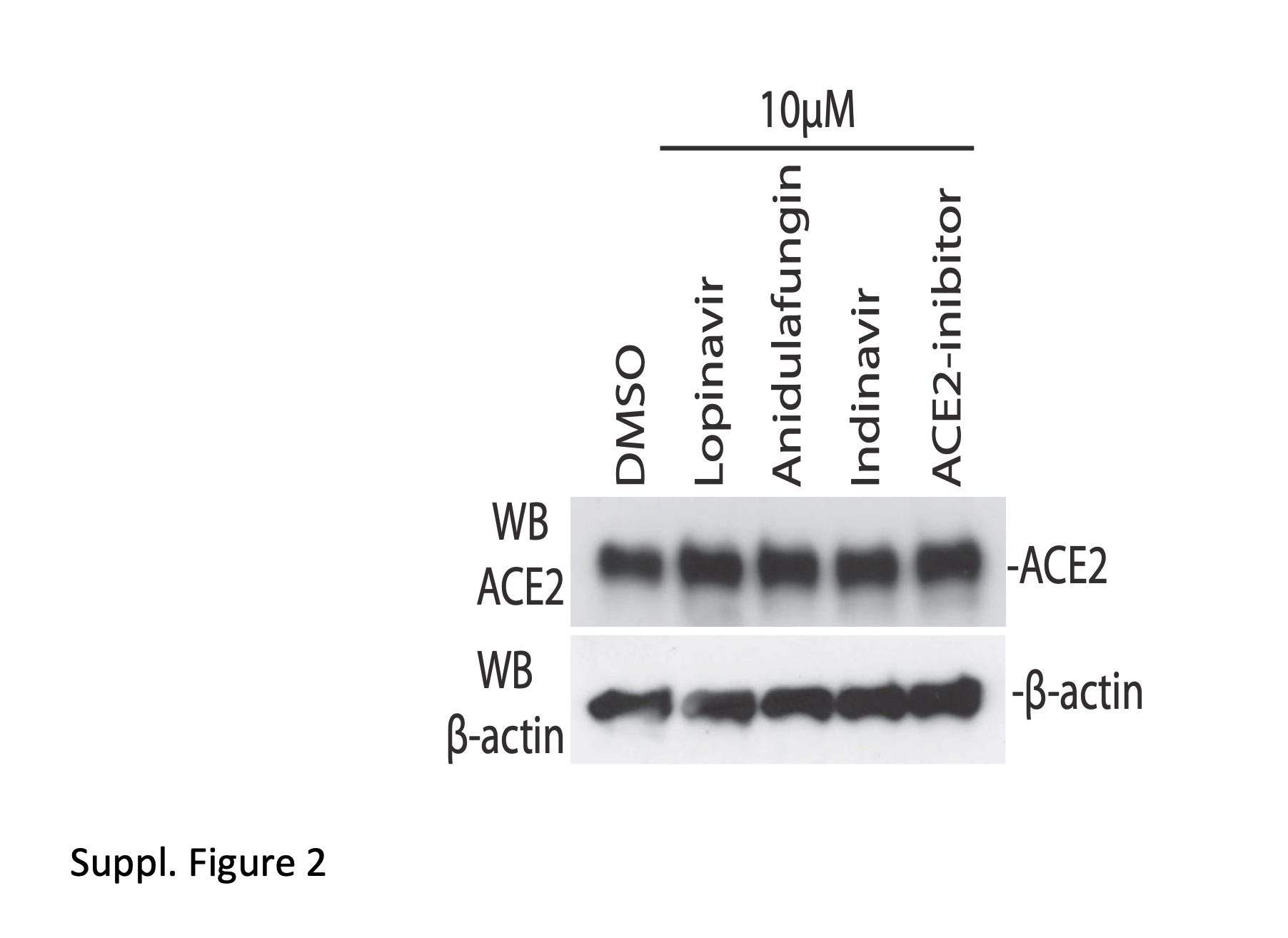

Supplement: Supplementary file 5 [file Image2.JPEG]
