# Supplementary material for: Anti-Fungal Drug Anidulafungin Inhibits SARS-CoV-2 Spike-Induced Syncytia Formation by Targeting ACE2-Spike Protein Interaction
Source: Front Genet. 2022 Mar 25;13:866474. doi: 10.3389/fgene.2022.866474 (PMC8990323; doi:10.3389/fgene.2022.866474)
Supplement: Supplementary file 2 [file Table1.docx]

**Table S1**. All the drugs along physicochemical properties and docking scores obtained from Flare software.

| **S. No.** | **PUBCHEM Compound CID** | **Radial Plot** | **MW** | **Atoms** | **SlogP** | **TPSA** | **Flexibility** | **RB** | **LF dG** | **LF VSscore** | **LF Rank Score** | **LF LE** |
| --- | --- | --- | --- | --- | --- | --- | --- | --- | --- | --- | --- | --- |
| 1 | 166548 | 0.25 | 1140.3 | 82 | 2.1 | 377.4 | 33.8 | 38 | -12.965 | -14.242 | -14.417 | -0.158 |
| 2 | 10074640 | 0.252 | 498.6 | 36 | 5.3 | 73.4 | 4 | 7 | -10.58 | -11.635 | -13.084 | -0.294 |
| 3 | 92727 | 0.037 | 628.8 | 46 | 4.7 | 120 | 16.5 | 16 | -8.773 | -11.057 | -12.973 | -0.191 |
| 4 | 5362440 | 0.188 | 613.8 | 45 | 3.6 | 118 | 14 | 14 | -8.937 | -10.565 | -12.939 | -0.199 |
| 5 | 3025960 | 0.25 | 609.2 | 44 | 6.4 | 72.2 | 8.7 | 11 | -10.538 | -12.353 | -12.926 | -0.239 |
| 6 | 60823 | 0.051 | 558.6 | 41 | 7.3 | 111.8 | 10.8 | 15 | -8.857 | -12.224 | -12.886 | -0.216 |
| 7 | 9852519 | 0.113 | 649.7 | 42 | 6.6 | 101.9 | 5.3 | 8 | -13.005 | -12.29 | -12.66 | -0.31 |
| 8 | 644241 | 0.14 | 529.5 | 39 | 6.4 | 97.6 | 1.3 | 7 | -8.413 | -11.082 | -12.607 | -0.216 |
| 9 | 208908 | 0.086 | 581.1 | 40 | 5.9 | 106.3 | 8 | 11 | -9.508 | -11.932 | -12.567 | -0.238 |
| 10 | 135449332 | 0.13 | 442.5 | 33 | 5.2 | 114.6 | 2 | 7 | -6.619 | -8.685 | -12.546 | -0.201 |
| 11 | 46220502 | 0.25 | 506.6 | 37 | 5.5 | 75 | 5.5 | 7 | -10.735 | -12.391 | -12.463 | -0.29 |
| 12 | 49843517 | 0.139 | 591.5 | 39 | 8.6 | 97.8 | 8.8 | 14 | -13.296 | -14.18 | -12.445 | -0.341 |
| 13 | 148192 | 0.05 | 704.9 | 51 | 4.6 | 171.2 | 15 | 21 | -9.263 | -11.398 | -12.408 | -0.182 |
| 14 | 11527774 | 0.25 | 533.1 | 38 | 7 | 65.8 | 7.3 | 7 | -9.109 | -10.935 | -12.404 | -0.24 |
| 15 | 5035 | 0.294 | 473.6 | 34 | 6.1 | 70 | 6 | 9 | -10.312 | -10.75 | -12.361 | -0.303 |
| 16 | 468595 | 0.051 | 700.8 | 51 | 6.2 | 111.8 | 10.7 | 13 | -8.68 | -11 | -12.353 | -0.17 |
| 17 | 392622 | 0 | 720.9 | 50 | 6.3 | 145.8 | 19 | 22 | -11.233 | -12.879 | -12.323 | -0.225 |
| 18 | 5717 | 0.027 | 575.7 | 41 | 5.7 | 115.7 | 6.5 | 10 | -8.465 | -9.681 | -12.188 | -0.206 |
| 19 | 5035 | 0.294 | 473.6 | 34 | 6.1 | 70 | 6 | 9 | -10.667 | -11.075 | -12.178 | -0.314 |
| 20 | 134827444 | 0.25 | 905.1 | 65 | -0.1 | 359.9 | 31 | 33 | -12.903 | -14.918 | -12.078 | -0.199 |
| 21 | 285033 | 0.213 | 545.6 | 39 | 3.3 | 124 | 11.8 | 11 | -8.578 | -10.024 | -11.982 | -0.22 |
| 22 | 50909779 | 0.271 | 487.6 | 36 | 1.9 | 120.1 | 11.5 | 12 | -7.782 | -9.952 | -11.975 | -0.216 |
| 23 | 9853053 | 0.25 | 693.7 | 50 | 8.4 | 61.4 | 9.2 | 12 | -8.252 | -12.149 | -11.921 | -0.165 |
| 24 | 24826799 | 0.313 | 532.6 | 39 | 4.5 | 65.8 | 4.5 | 7 | -7.071 | -9.618 | -11.854 | -0.181 |
| 25 | 11103 | 0 | 598.7 | 44 | 7.4 | 172.4 | 10.5 | 12 | -9.409 | -12.045 | -11.826 | -0.214 |
| 26 | 20055152 | 0.362 | 420.5 | 31 | 4.9 | 85.2 | 1.6 | 3 | -8.18 | -10.134 | -11.758 | -0.264 |
| 27 | 9826520 | 0.28 | 482 | 33 | 5.9 | 54.4 | 4.9 | 7 | -7.545 | -8.69 | -11.758 | -0.229 |
| 28 | 10316203 | 0.49 | 355.9 | 25 | 5.3 | 57.2 | 3 | 6 | -9.74 | -10.97 | -11.732 | -0.39 |
| 29 | 154257 | 0.299 | 470.6 | 35 | 6.3 | 57.9 | 8.6 | 9 | -11.051 | -12.58 | -11.722 | -0.316 |
| 30 | 153241 | 0.279 | 452.5 | 33 | 3.4 | 123.9 | 5.5 | 11 | -8.378 | -10.44 | -11.717 | -0.254 |
| 31 | 42890 | 0.254 | 497.5 | 36 | 1.8 | 176.6 | 8.4 | 8 | -6.969 | -10.618 | -11.711 | -0.194 |
| 32 | 11511120 | 0.3 | 469.9 | 33 | 5.3 | 79.4 | 4.5 | 7 | -9.884 | -11.139 | -11.683 | -0.3 |
| 33 | 275182 | 0.25 | 608.7 | 45 | 6.9 | 72.9 | 4 | 4 | -9.045 | -10.296 | -11.623 | -0.201 |
| 34 | 24873435 | 0 | 749.9 | 52 | 5.1 | 156.9 | 12 | 8 | -10.777 | -11.724 | -11.621 | -0.207 |
| 35 | 71447 | 0.281 | 481.5 | 34 | 1.6 | 128 | 7.4 | 6 | -7.605 | -8.865 | -11.615 | -0.224 |
| 36 | 3961 | 0.338 | 422.9 | 30 | 4.7 | 92.5 | 7 | 9 | -7.479 | -8.679 | -11.591 | -0.249 |
| 37 | 658293 | 0.548 | 373.5 | 28 | 4.3 | 59.8 | 7.5 | 8 | -5.215 | -7.277 | -11.583 | -0.186 |
| 38 | 275182 | 0.25 | 608.7 | 45 | 6.9 | 72.9 | 4 | 4 | -8.726 | -10.153 | -11.529 | -0.194 |
| 39 | 95168 | 0.409 | 404.5 | 30 | 5.2 | 76 | 3.2 | 5 | -8.041 | -9.096 | -11.448 | -0.268 |
| 40 | 24748573 | 0.512 | 349.8 | 25 | 4.9 | 75.1 | 1 | 4 | -5.533 | -7.518 | -11.44 | -0.221 |
| 41 | 10624680 | 0.285 | 483.6 | 36 | 4.2 | 94.7 | 2.2 | 6 | -9.442 | -10.829 | -11.347 | -0.262 |
| 42 | 11282283 | 0.588 | 447.5 | 32 | 2.7 | 75.9 | 3.7 | 5 | -7.212 | -8.445 | -11.309 | -0.225 |
| 43 | 25151352 | 0.387 | 417.8 | 29 | 5.3 | 66.5 | 4.3 | 6 | -6.951 | -9.721 | -11.204 | -0.24 |
| 44 | 119171 | 0 | 631.7 | 45 | 7.2 | 122.9 | 6.7 | 10 | -7.988 | -10.461 | -11.152 | -0.178 |
| 45 | 131411 | 0.288 | 477.4 | 29 | 5.3 | 54.7 | 6.8 | 8 | -11.07 | -11.82 | -11.139 | -0.382 |
| 46 | 49806644 | 0 | 660.5 | 47 | 6.8 | 129.3 | 8 | 14 | -6.228 | -10.613 | -11.117 | -0.133 |
| 47 | 2247 | 0.255 | 458.6 | 34 | 5.6 | 42.3 | 7.7 | 8 | -9.389 | -11.203 | -11.086 | -0.276 |
| 48 | 3062316 | 0.267 | 488 | 33 | 3.7 | 106.5 | 5.2 | 8 | -8.782 | -9.938 | -11.061 | -0.266 |
| 49 | 9893640 | 0.25 | 544.8 | 38 | 6.3 | 54.4 | 10 | 13 | -11.147 | -12.278 | -11.039 | -0.293 |
| 50 | 16722836 | 0.097 | 524.7 | 37 | 4.8 | 108.5 | 6.5 | 11 | -9.367 | -10.116 | -11.026 | -0.253 |
| 51 | 2585 | 0.518 | 406.5 | 30 | 4.1 | 75.7 | 9.5 | 11 | -7.672 | -9.134 | -10.994 | -0.256 |
| 52 | 68060125 | 0.25 | 513.4 | 35 | 5.3 | 73.4 | 2.9 | 5 | -8.192 | -9.761 | -10.987 | -0.234 |
| 53 | 16720766 | 0.344 | 443.5 | 31 | 2.4 | 132.4 | 6.3 | 8 | -10.093 | -11.963 | -10.98 | -0.326 |
| 54 | 6442177 | 0 | 958.2 | 68 | 6.9 | 204.7 | 32.9 | 33 | -13.607 | -14.183 | -10.954 | -0.2 |
| 55 | 148121 | 0.287 | 477.5 | 35 | 1.1 | 207.3 | 9.8 | 12 | -10.607 | -12.788 | -10.904 | -0.303 |
| 56 | 216239 | 0.232 | 464.8 | 32 | 5.6 | 92.3 | 2.3 | 8 | -5.754 | -8.278 | -10.902 | -0.18 |
| 57 | 9887537 | 0.393 | 405.5 | 30 | 4.3 | 37.8 | 3.7 | 4 | -7.182 | -8.885 | -10.9 | -0.239 |
| 58 | 3220 | 0.657 | 270.2 | 20 | 1.9 | 94.8 | 1.5 | 3 | -5.978 | -7.573 | -10.866 | -0.299 |
| 59 | 208917 | 0.386 | 375.4 | 28 | 4.6 | 35.4 | 3.2 | 4 | -4.843 | -7.003 | -10.862 | -0.173 |
| 60 | 656630 | 0.175 | 985.1 | 69 | 3.6 | 288.3 | 24.7 | 19 | -10.168 | -16.675 | -10.81 | -0.147 |
| 61 | 126941 | 0.326 | 454.4 | 33 | 0.3 | 210.5 | 8 | 11 | -7.696 | -11.824 | -10.777 | -0.233 |
| 62 | 6536 | 0.209 | 438 | 31 | 6.3 | 32.7 | 10.5 | 11 | -7.83 | -9.803 | -10.757 | -0.253 |
| 63 | 71315012 | 0.25 | 544.5 | 39 | 1.9 | 189.3 | 11.2 | 16 | -7.712 | -11.173 | -10.727 | -0.198 |
| 64 | 64143 | 0.113 | 567.8 | 40 | 5.1 | 101.9 | 11.5 | 12 | -9.327 | -10.424 | -10.726 | -0.233 |
| 65 | 3955 | 0.237 | 477 | 34 | 5.5 | 43.8 | 8.2 | 9 | -7.153 | -8.975 | -10.724 | -0.21 |
| 66 | 25033539 | 0.374 | 425.5 | 31 | 2.4 | 123.7 | 5.8 | 7 | -7.712 | -9.497 | -10.719 | -0.249 |
| 67 | 133633 | 0.448 | 340.9 | 24 | 4.7 | 39.3 | 4 | 4 | -8.001 | -8.477 | -10.645 | -0.333 |
| 68 | 5282209 | 0.103 | 590.7 | 43 | 4.2 | 119.5 | 7.1 | 8 | -9.062 | -10.377 | -10.639 | -0.211 |
| 69 | 5388906 | 0.213 | 555.5 | 37 | 7.6 | 45.6 | 8.5 | 9 | -6.285 | -8.791 | -10.637 | -0.17 |
| 70 | 11167602 | 0.202 | 482.8 | 33 | 6 | 92.3 | 2.3 | 8 | -5.482 | -8.324 | -10.628 | -0.166 |
| 71 | 3396 | 0.171 | 475.6 | 35 | 5.8 | 35.6 | 7.7 | 7 | -7.663 | -10.393 | -10.619 | -0.219 |
| 72 | 24775005 | 0.274 | 485.5 | 35 | 5.8 | 63.7 | 2.2 | 6 | -8.448 | -10.895 | -10.593 | -0.241 |
| 73 | 208836 | 0.313 | 375.5 | 28 | 5.2 | 32.7 | 10 | 10 | -7.56 | -8.619 | -10.59 | -0.27 |
| 74 | 25262965 | 0.475 | 465.6 | 34 | 3 | 93.1 | 4.9 | 6 | -6.576 | -9.047 | -10.583 | -0.193 |
| 75 | 135465539 | 0.55 | 364.4 | 27 | 3.2 | 104.2 | 5 | 6 | -10.37 | -13.165 | -10.577 | -0.384 |
| 76 | 3926 | 0.144 | 491.6 | 36 | 6.3 | 35.6 | 9.2 | 9 | -9.629 | -11.482 | -10.549 | -0.267 |
| 77 | 14982 | 0.113 | 822.9 | 58 | 4.1 | 267 | 18.2 | 15 | -8.636 | -15.514 | -10.53 | -0.149 |
| 78 | 2165 | 0.477 | 355.9 | 25 | 5.2 | 48.4 | 5 | 7 | -8.679 | -9.108 | -10.482 | -0.347 |
| 79 | 150311 | 0.401 | 409.4 | 30 | 5.8 | 60.8 | 5.8 | 8 | -7.854 | -8.985 | -10.431 | -0.262 |
| 80 | 9417 | 0.473 | 363.5 | 27 | 3.7 | 29.9 | 8 | 7 | -9.126 | -10.478 | -10.417 | -0.338 |
| 81 | 69879809 | 0.612 | 368.4 | 27 | 3.6 | 85.2 | 3 | 7 | -8.408 | -9.35 | -10.412 | -0.311 |
| 82 | 446155 | 0.381 | 411.5 | 30 | 5.6 | 82.7 | 8.5 | 11 | -7.166 | -10.506 | -10.39 | -0.239 |
| 83 | 11978813 | 0.342 | 433.6 | 31 | 4.8 | 44.8 | 6.9 | 7 | -8.231 | -9.182 | -10.343 | -0.266 |
| 84 | 16779 | 0.414 | 401.5 | 22 | 5.7 | 69.6 | 1.5 | 4 | -8.109 | -9.227 | -10.339 | -0.369 |
| 85 | 22049997 | 0.288 | 477.3 | 30 | 5.2 | 58.6 | 3.2 | 5 | -6.685 | -8.211 | -10.325 | -0.223 |
| 86 | 129738071 | 0.508 | 382.4 | 28 | 2.7 | 110.1 | 5 | 10 | -8.943 | -10.003 | -10.311 | -0.319 |
| 87 | 6436173 | 0 | 785.9 | 57 | 6.5 | 198.4 | 16.5 | 20 | -9.73 | -11.013 | -10.304 | -0.171 |
| 88 | 53469448 | 0.406 | 451.4 | 33 | 4.4 | 77 | 2.3 | 5 | -5.855 | -8.528 | -10.3 | -0.177 |
| 89 | 5497186 | 0.518 | 414.5 | 31 | 4 | 68.7 | 5 | 4 | -8.988 | -9.429 | -10.281 | -0.29 |
| 90 | 3033767 | 0.293 | 387.5 | 29 | 5.7 | 32.7 | 5.8 | 9 | -9.029 | -9.887 | -10.28 | -0.311 |
| 91 | 5287969 | 0.537 | 401.8 | 28 | 3.5 | 90.2 | 3 | 5 | -7.418 | -8.436 | -10.239 | -0.265 |
| 92 | 16220172 | 0.429 | 392.5 | 29 | 5 | 78.4 | 1 | 5 | -6.546 | -8.017 | -10.159 | -0.226 |
| 93 | 439201 | 0.25 | 1060.2 | 76 | -3.6 | 418.8 | 32 | 33 | -11.539 | -14.562 | -10.157 | -0.152 |
| 94 | 44129660 | 0.421 | 418.4 | 30 | 3.9 | 96.4 | 4.3 | 9 | -6.425 | -8.715 | -10.132 | -0.214 |
| 95 | 5282219 | 0.319 | 450.5 | 33 | 5.5 | 82.1 | 8.5 | 13 | -6.718 | -8.705 | -10.123 | -0.204 |
| 96 | 10047612 | 0.165 | 418.4 | 28 | 6.6 | 23.5 | 5.2 | 4 | -7.649 | -8.921 | -10.09 | -0.273 |
| 97 | 122019 | 0.661 | 386.4 | 28 | 2.6 | 84.5 | 9.5 | 11 | -7.161 | -7.631 | -10.073 | -0.256 |
| 98 | 6436090 | 0.379 | 461.5 | 33 | 3.2 | 105.7 | 1.7 | 5 | -7.118 | -8.508 | -10.066 | -0.216 |
| 99 | 3949 | 0.171 | 468.5 | 34 | 5.4 | 34.2 | 7.5 | 8 | -8.32 | -10.261 | -10.012 | -0.245 |
| 100 | 5315472 | 0.725 | 308.3 | 23 | 3.2 | 74.6 | 4 | 8 | -6.141 | -7.726 | -10.008 | -0.267 |
| 101 | 5284613 | 0.113 | 530.7 | 38 | 4.1 | 125.7 | 9.4 | 7 | -8.498 | -11.52 | -10.007 | -0.224 |
| 102 | 9864510 | 0.579 | 377.5 | 27 | 4 | 74.1 | 5.3 | 6 | -6.417 | -8.279 | -9.945 | -0.238 |
| 103 | 1715 | 0.295 | 424.6 | 30 | 4.5 | 32.8 | 7.9 | 7 | -6.922 | -7.908 | -9.938 | -0.231 |
| 104 | 467825 | 0.307 | 437.5 | 31 | 5.2 | 87.6 | 7 | 7 | -9.075 | -10.388 | -9.901 | -0.293 |
| 105 | 91769 | 0.343 | 381.5 | 27 | 5.2 | 37.4 | 7.7 | 8 | -7.417 | -8.333 | -9.875 | -0.275 |
| 106 | 11068834 | 0.51 | 376.4 | 27 | 3.6 | 99.4 | 12 | 14 | -9.592 | -10.07 | -9.868 | -0.355 |
| 107 | 704473 | 0.486 | 267.3 | 19 | 4.3 | 37.8 | 0.5 | 3 | -5.698 | -6.893 | -9.867 | -0.3 |
| 108 | 3372 | 0.224 | 437.5 | 30 | 4.7 | 29.9 | 8 | 8 | -8.489 | -10.499 | -9.859 | -0.283 |
| 109 | 3372 | 0.224 | 437.5 | 30 | 4.7 | 29.9 | 8 | 8 | -8.648 | -10.961 | -9.845 | -0.288 |
| 110 | 2724385 | 0.075 | 780.9 | 55 | 4.4 | 203.1 | 17.8 | 13 | -9.966 | -13.655 | -9.838 | -0.181 |
| 111 | 19675 | 0.347 | 410.6 | 29 | 5.4 | 43.8 | 8.2 | 8 | -7.152 | -8.907 | -9.838 | -0.247 |
| 112 | 5803 | 0.25 | 621.9 | 21 | 5.4 | 66.8 | 3.8 | 6 | -7.72 | -9.075 | -9.789 | -0.368 |
| 113 | 6918155 | 0.143 | 540.7 | 39 | 4.9 | 99.1 | 8.1 | 6 | -9.506 | -9.758 | -9.785 | -0.244 |
| 114 | 448281 | 0.417 | 428.3 | 28 | 3.4 | 104.4 | 10.5 | 12 | -11.113 | -11.467 | -9.783 | -0.397 |
| 115 | 42611257 | 0.192 | 489.9 | 33 | 6.1 | 91.9 | 3.3 | 7 | -6.344 | -8.468 | -9.77 | -0.192 |
| 116 | 5318039 | 0.558 | 374.4 | 27 | 3.4 | 96.2 | 11 | 13 | -7.741 | -9.317 | -9.763 | -0.287 |
| 117 | 121304016 | 0.237 | 602.6 | 42 | 3.1 | 203.5 | 15 | 15 | -3.454 | -9.487 | -9.759 | -0.082 |
| 118 | 119146 | 0.5 | 409.5 | 30 | 3.8 | 43.9 | 6 | 6 | -5.658 | -7.448 | -9.752 | -0.189 |
| 119 | 5281078 | 0.52 | 433.5 | 31 | 2.5 | 94.5 | 10 | 10 | -9.282 | -10.275 | -9.751 | -0.299 |
| 120 | 33630 | 0.029 | 524 | 36 | 8.5 | 23.5 | 8 | 9 | -7.544 | -10.079 | -9.749 | -0.21 |
| 121 | 11977753 | 0.301 | 469.5 | 36 | 7 | 73.1 | 2.5 | 3 | -10.086 | -13.304 | -9.742 | -0.28 |
| 122 | 3002977 | 0.25 | 513.7 | 37 | 6 | 63 | 9.1 | 8 | -6.721 | -9.059 | -9.739 | -0.182 |
| 123 | 5469424 | 0.701 | 338.4 | 25 | 3.2 | 83.8 | 4.5 | 9 | -7.803 | -8.919 | -9.734 | -0.312 |
| 124 | 4747 | 0.549 | 365.5 | 26 | 4.4 | 50.5 | 7 | 5 | -7.389 | -8.647 | -9.729 | -0.284 |
| 125 | 71739 | 0.7 | 298.4 | 22 | 3.4 | 57.3 | 6.5 | 7 | -8.591 | -9.381 | -9.637 | -0.39 |
| 126 | 51045 | 0.621 | 363.5 | 27 | 3.8 | 81.1 | 9.5 | 9 | -8.714 | -9.45 | -9.635 | -0.323 |
| 127 | 276389 | 0.25 | 531.6 | 38 | 2.9 | 124 | 10.8 | 10 | -9.325 | -9.939 | -9.632 | -0.245 |
| 128 | 25182468 | 0.298 | 549.5 | 34 | 4.2 | 88.3 | 13.5 | 14 | -9.85 | -10.598 | -9.63 | -0.29 |
| 129 | 33887 | 0.35 | 477.6 | 35 | 4.5 | 69.2 | 8.7 | 10 | -7.618 | -10.028 | -9.621 | -0.218 |
| 130 | 6918289 | 0 | 1030.3 | 73 | 6.8 | 242 | 34.9 | 35 | -12.897 | -14.483 | -9.617 | -0.177 |
| 131 | 439530 | 0.297 | 471.5 | 34 | 0 | 160.9 | 9.4 | 11 | -6.169 | -8.599 | -9.61 | -0.181 |
| 132 | 9913767 | 0.259 | 496.6 | 35 | 3.9 | 101.5 | 8.8 | 12 | -9.099 | -9.745 | -9.596 | -0.26 |
| 133 | 11539025 | 0.349 | 420.5 | 31 | 5.8 | 85.4 | 1 | 5 | -8.368 | -9.72 | -9.593 | -0.27 |
| 134 | 58298316 | 0.543 | 397.5 | 29 | 2.9 | 100.5 | 4.6 | 5 | -8.034 | -9.014 | -9.564 | -0.277 |
| 135 | 5440 | 0.167 | 399.6 | 27 | 5 | 9.7 | 7 | 6 | -10.037 | -10.532 | -9.551 | -0.372 |
| 136 | 9916461 | 0.25 | 587.9 | 41 | 7.4 | 73.6 | 2.5 | 8 | -5.31 | -8.943 | -9.547 | -0.13 |
| 137 | 441207 | 0 | 764.9 | 54 | 5.1 | 182.8 | 16.8 | 12 | -7.365 | -14.296 | -9.546 | -0.136 |
| 138 | 3085092 | 0 | 751 | 53 | 7.2 | 161.2 | 18.1 | 16 | -11.108 | -12.685 | -9.52 | -0.21 |
| 139 | 82146 | 0.394 | 348.5 | 26 | 6 | 37.3 | 1.5 | 4 | -7.158 | -8.773 | -9.509 | -0.275 |
| 140 | 5978 | 0.113 | 825 | 60 | 4.1 | 171.2 | 12 | 10 | -7.057 | -10.482 | -9.508 | -0.118 |
| 141 | 9429 | 0.54 | 446.6 | 30 | 3.2 | 47.1 | 7 | 6 | -10.002 | -10.562 | -9.489 | -0.333 |
| 142 | 10429233 | 0.609 | 370.4 | 27 | 3.2 | 93.1 | 7.5 | 11 | -8.483 | -9.258 | -9.481 | -0.314 |
| 143 | 219025 | 0.195 | 556.8 | 39 | 6.5 | 88.8 | 14.8 | 18 | -9.055 | -10.334 | -9.468 | -0.232 |
| 144 | 54892 | 0.503 | 438.5 | 32 | 2.6 | 95.9 | 10 | 11 | -7.412 | -9.031 | -9.464 | -0.232 |
| 145 | 71657455 | 0.054 | 583 | 41 | 4.6 | 119.3 | 7.3 | 8 | -7.79 | -10.096 | -9.435 | -0.19 |
| 146 | 5381 | 0.482 | 351.5 | 25 | 4.4 | 39.2 | 2.7 | 4 | -8.403 | -9.048 | -9.411 | -0.336 |
| 147 | 5440 | 0.167 | 399.6 | 27 | 5 | 9.7 | 7 | 6 | -10.596 | -10.87 | -9.386 | -0.392 |
| 148 | 208898 | 0.195 | 556.8 | 39 | 6.5 | 88.8 | 14.8 | 18 | -8.107 | -10.144 | -9.355 | -0.208 |
| 149 | 53315106 | 0.237 | 574.8 | 41 | 8.7 | 82.1 | 19 | 19 | -9.297 | -10.394 | -9.345 | -0.227 |
| 150 | 387447 | 0.443 | 384.2 | 28 | 0.8 | 124.4 | 10.3 | 11 | -6.28 | -8.188 | -9.336 | -0.224 |
| 151 | 124072 | 0.606 | 372.4 | 27 | 3.2 | 93.1 | 10 | 12 | -7.82 | -8.265 | -9.322 | -0.29 |
| 152 | 3598 | 0.326 | 406.9 | 21 | 6.8 | 40.5 | 3 | 4 | -7.578 | -9.002 | -9.314 | -0.361 |
| 153 | 9876378 | 0 | 966.2 | 69 | 6.4 | 218.8 | 28.9 | 30 | -11.432 | -12.685 | -9.212 | -0.166 |
| 154 | 71301 | 0.57 | 405.4 | 29 | 3.7 | 70.9 | 9 | 8 | -10.195 | -11.653 | -9.197 | -0.352 |
| 155 | 439501 | 0.25 | 584.7 | 41 | 1.5 | 206.6 | 14.9 | 12 | -8.99 | -11.127 | -9.116 | -0.219 |
| 156 | 5284587 | 0.639 | 372.5 | 27 | 2.6 | 91.7 | 5.8 | 4 | -6.937 | -8.646 | -9.086 | -0.257 |
| 157 | 10445549 | 0.5 | 265.3 | 19 | -0.6 | 140.3 | 4.5 | 5 | -8.643 | -8.316 | -9.08 | -0.455 |
| 158 | 6741 | 0.617 | 374.5 | 27 | 2.7 | 94.8 | 6.8 | 5 | -8.09 | -9.054 | -9.03 | -0.3 |
| 159 | 5311510 | 0.534 | 374.3 | 27 | 3.9 | 90.1 | 1.3 | 6 | -4.977 | -7.96 | -9.025 | -0.184 |
| 160 | 77254 | 0.594 | 326.6 | 18 | 4.2 | 49.3 | 1 | 3 | -4.882 | -6.506 | -9.021 | -0.271 |
| 161 | 941361 | 0.159 | 404.5 | 30 | 5.9 | 6.5 | 6.2 | 6 | -7.15 | -9.304 | -9.017 | -0.238 |
| 162 | 68186 | 0.613 | 383.5 | 28 | 3.3 | 44.8 | 6.2 | 6 | -5.839 | -7.256 | -9.014 | -0.209 |
| 163 | 119607 | 0.711 | 314.4 | 22 | 2.4 | 86.2 | 2 | 4 | -4.675 | -5.959 | -9.008 | -0.213 |
| 164 | 45375808 | 0.25 | 529.5 | 36 | 2.2 | 152.7 | 10.5 | 11 | -4.305 | -10.981 | -9.007 | -0.12 |
| 165 | 108143 | 0.498 | 351.4 | 26 | 5 | 66.4 | 1.5 | 4 | -7.32 | -8.617 | -8.997 | -0.282 |
| 166 | 444025 | 0.298 | 467 | 32 | 4.1 | 99.1 | 7.3 | 7 | -5.93 | -7.661 | -8.992 | -0.185 |
| 167 | 2662 | 0.635 | 381.4 | 26 | 3.5 | 78 | 2.3 | 5 | -5.694 | -9.459 | -8.973 | -0.219 |
| 168 | 5312149 | 0.443 | 452 | 29 | 4.1 | 70.7 | 3.5 | 5 | -7.261 | -8.116 | -8.958 | -0.25 |
| 169 | 5566 | 0.167 | 407.5 | 28 | 4.9 | 9.7 | 5 | 5 | -6.422 | -7.939 | -8.948 | -0.229 |
| 170 | 6075 | 0.3 | 310.5 | 22 | 4.6 | 6.5 | 3 | 2 | -5.411 | -6.468 | -8.939 | -0.246 |
| 171 | 60835 | 0.311 | 297.4 | 21 | 4.6 | 21.3 | 5.5 | 6 | -7.786 | -8.517 | -8.914 | -0.371 |
| 172 | 5452 | 0.216 | 370.6 | 25 | 5.9 | 6.5 | 5 | 4 | -9.326 | -9.764 | -8.909 | -0.373 |
| 173 | 4139 | 0.5 | 284.4 | 20 | 1.9 | 18.6 | 0.5 | 1 | -6.702 | -7.224 | -8.88 | -0.335 |
| 174 | 4066 | 0.3 | 322.5 | 23 | 4.6 | 6.5 | 3.4 | 2 | -6.475 | -7.383 | -8.874 | -0.282 |
| 175 | 446541 | 0.668 | 320.3 | 23 | 2.7 | 93.1 | 6.3 | 8 | -7.345 | -8.99 | -8.849 | -0.319 |
| 176 | 23652732 | 0.486 | 418.6 | 30 | 4.2 | 71 | 14 | 13 | -9.328 | -10.413 | -8.79 | -0.311 |
| 177 | 166558 | 0.25 | 501.7 | 35 | 7.3 | 72.5 | 8.5 | 10 | -7.674 | -8.859 | -8.787 | -0.219 |
| 178 | 10071196 | 0.34 | 427.6 | 31 | 4.9 | 44.8 | 9 | 10 | -6.561 | -8.295 | -8.781 | -0.212 |
| 179 | 479503 | 0.657 | 288.3 | 21 | 2.4 | 94.8 | 5 | 6 | -7.404 | -7.848 | -8.773 | -0.353 |
| 180 | 5288783 | 0.396 | 412.6 | 30 | 5.9 | 60.7 | 9.5 | 8 | -10.036 | -10.356 | -8.749 | -0.335 |
| 181 | 54726191 | 0.514 | 419.4 | 30 | 1.6 | 99.2 | 4.2 | 4 | -8.141 | -10.428 | -8.691 | -0.271 |
| 182 | 14677 | 0.35 | 296.4 | 21 | 4.2 | 6.5 | 2.5 | 2 | -5.297 | -6.251 | -8.68 | -0.252 |
| 183 | 155794 | 0.637 | 329.4 | 23 | 3.9 | 60 | 4 | 5 | -9.235 | -9.704 | -8.669 | -0.402 |
| 184 | 6410104 | 0.677 | 317.4 | 24 | 3 | 91.7 | 1 | 3 | -9.108 | -12.74 | -8.667 | -0.379 |
| 185 | 159516 | 0.309 | 464.6 | 34 | 6.6 | 63.6 | 3.7 | 2 | -8.334 | -8.854 | -8.661 | -0.245 |
| 186 | 1238 | 0.325 | 344.9 | 23 | 4.4 | 6.5 | 1.3 | 1 | -6.705 | -7.707 | -8.659 | -0.292 |
| 187 | 5284616 | 0 | 914.2 | 65 | 6.9 | 195.4 | 29.9 | 30 | -10.331 | -11.221 | -8.594 | -0.159 |
| 188 | 969494 | 0.25 | 421.5 | 29 | 4.5 | 26.8 | 4.2 | 5 | -5.658 | -7.995 | -8.593 | -0.195 |
| 189 | 91503 | 0.613 | 326.4 | 24 | 3.8 | 45.6 | 5.9 | 5 | -7.095 | -8.117 | -8.51 | -0.296 |
| 190 | 4046 | 0.437 | 378.3 | 26 | 4.8 | 45.1 | 4 | 5 | -6.141 | -9.089 | -8.502 | -0.236 |
| 191 | 3728 | 0.443 | 397 | 13 | 3.7 | 33.1 | 0.5 | 1 | -5.722 | -6.622 | -8.493 | -0.44 |
| 192 | 445154 | 0.75 | 228.2 | 17 | 3 | 60.7 | 2 | 5 | -7.215 | -7.36 | -8.474 | -0.424 |
| 193 | 46215462 | 0.151 | 506.7 | 38 | 4.9 | 97.8 | 3.1 | 4 | -9.155 | -12.668 | -8.465 | -0.241 |
| 194 | 44462760 | 0.057 | 519.6 | 35 | 6.2 | 110.9 | 1.8 | 6 | -6.874 | -8.722 | -8.46 | -0.196 |
| 195 | 3652 | 0.587 | 335.9 | 23 | 4.2 | 48.4 | 9.3 | 10 | -8.689 | -10.048 | -8.425 | -0.378 |
| 196 | 4477 | 0.555 | 327.1 | 21 | 3.8 | 95.2 | 1.3 | 4 | -5.174 | -6.721 | -8.417 | -0.246 |
| 197 | 9865442 | 0.614 | 394.9 | 27 | 3.3 | 83.8 | 5.8 | 4 | -6.681 | -7.616 | -8.415 | -0.247 |
| 198 | 5475158 | 0.44 | 340.5 | 24 | 4.3 | 32.3 | 5.8 | 8 | -6.198 | -7.342 | -8.415 | -0.258 |
| 199 | 5281104 | 0.389 | 416.6 | 30 | 6.4 | 60.7 | 9.5 | 8 | -10.27 | -10.202 | -8.408 | -0.342 |
| 200 | 667467 | 0.25 | 315.9 | 21 | 5.1 | 3.2 | 3 | 3 | -6.98 | -7.569 | -8.393 | -0.332 |
| 201 | 5284571 | 0.5 | 280.3 | 21 | 2.1 | 120 | 1.5 | 5 | -7.49 | -8.623 | -8.392 | -0.357 |
| 202 | 441074 | 0.663 | 324.4 | 24 | 3.4 | 45.6 | 5.9 | 5 | -7.036 | -8.031 | -8.387 | -0.293 |
| 203 | 21071390 | 0.311 | 398.4 | 28 | 5.2 | 37 | 8.5 | 9 | -6.514 | -9.246 | -8.373 | -0.233 |
| 204 | 2519269 | 0.66 | 394.5 | 26 | 2.6 | 82.6 | 3.3 | 5 | -5.966 | -6.924 | -8.355 | -0.229 |
| 205 | 4632 | 0.721 | 228.2 | 17 | 2.6 | 46.5 | 1.5 | 4 | -4.925 | -5.929 | -8.288 | -0.29 |
| 206 | 5351307 | 0.671 | 234.3 | 16 | 1.4 | 40.5 | 1.3 | 4 | -11.105 | -11.278 | -8.269 | -0.694 |
| 207 | 444029 | 0.25 | 509.6 | 36 | 2.6 | 122.6 | 10.6 | 8 | -9.402 | -10.343 | -8.269 | -0.261 |
| 208 | 5572 | 0.317 | 301.5 | 22 | 4.7 | 23.5 | 7.9 | 6 | -9.1 | -9.314 | -8.268 | -0.414 |
| 209 | 2663 | 0.595 | 379.5 | 27 | 3.3 | 90.9 | 8.5 | 13 | -9.79 | -10.564 | -8.254 | -0.363 |
| 210 | 65814 | 0.604 | 292.2 | 18 | 3.6 | 41.5 | 5.5 | 7 | -9.371 | -9.677 | -8.215 | -0.521 |
| 211 | 159501 | 0.75 | 316.3 | 23 | 2.6 | 67.4 | 1 | 1 | -5.701 | -6.888 | -8.212 | -0.248 |
| 212 | 3929516 | 0.25 | 343.4 | 25 | 5.2 | 12.5 | 5.1 | 4 | -7.374 | -7.851 | -8.197 | -0.295 |
| 213 | 5473838 | 0.326 | 438.7 | 32 | 6.5 | 46.9 | 11.5 | 14 | -8.717 | -9.403 | -8.195 | -0.272 |
| 214 | 41684 | 0.537 | 307.3 | 21 | 2.1 | 114.1 | 1.3 | 4 | -5.062 | -6.205 | -8.191 | -0.241 |
| 215 | 5362119 | 0.407 | 405.5 | 29 | 1.2 | 133 | 14.3 | 16 | -10.154 | -10.342 | -8.173 | -0.35 |
| 216 | 11681588 | 0.563 | 337.4 | 25 | 4.5 | 69.6 | 1 | 3 | -5.812 | -7.166 | -8.138 | -0.232 |
| 217 | 10334137 | 0.75 | 285.4 | 20 | 1.7 | 53.4 | 1.5 | 5 | -9.839 | -10.178 | -8.12 | -0.492 |
| 218 | 60657 | 0.75 | 307.4 | 22 | 2.8 | 50.7 | 11.5 | 12 | -9.523 | -10.09 | -8.1 | -0.433 |
| 219 | 441145 | 0 | 670.9 | 47 | 5.4 | 153.4 | 13.9 | 14 | -7.177 | -11.745 | -8.098 | -0.153 |
| 220 | 3386 | 0.336 | 309.3 | 22 | 4.4 | 21.3 | 5.5 | 7 | -7.885 | -9.215 | -8.096 | -0.358 |
| 221 | 13298169 | 0.557 | 416.5 | 30 | 2.9 | 93.1 | 7.2 | 5 | -7.097 | -8.307 | -8.09 | -0.237 |
| 222 | 5291 | 0.271 | 493.6 | 37 | 4.6 | 86.3 | 4 | 7 | -8.127 | -11.106 | -8.056 | -0.22 |
| 223 | 441074 | 0.663 | 324.4 | 24 | 3.4 | 45.6 | 5.9 | 5 | -7.258 | -7.855 | -8.037 | -0.302 |
| 224 | 135398508 | 0.5 | 277.3 | 20 | -0.3 | 125.8 | 3.8 | 4 | -7.737 | -7.915 | -8.036 | -0.387 |
| 225 | 5574 | 0.313 | 298.4 | 21 | 4.5 | 6.5 | 4 | 4 | -8.149 | -8.654 | -8.012 | -0.388 |
| 226 | 3386 | 0.336 | 309.3 | 22 | 4.4 | 21.3 | 5.5 | 7 | -7.831 | -9.18 | -7.982 | -0.356 |
| 227 | 5291 | 0.271 | 493.6 | 37 | 4.6 | 86.3 | 4 | 7 | -8.448 | -11.193 | -7.979 | -0.228 |
| 228 | 4927 | 0.35 | 284.4 | 20 | 4.2 | 6.5 | 3 | 3 | -5.109 | -5.999 | -7.975 | -0.255 |
| 229 | 72287 | 0.313 | 328.5 | 23 | 4.5 | 15.7 | 4.5 | 5 | -6.069 | -6.722 | -7.971 | -0.264 |
| 230 | 4927 | 0.35 | 284.4 | 20 | 4.2 | 6.5 | 3 | 3 | -4.778 | -5.776 | -7.966 | -0.239 |
| 231 | 5283731 | 0.336 | 400.6 | 29 | 7.3 | 40.5 | 9.5 | 8 | -9.55 | -10.174 | -7.892 | -0.329 |
| 232 | 107778 | 0.436 | 386.6 | 28 | 6.5 | 49.7 | 9.3 | 9 | -7.292 | -8.847 | -7.891 | -0.26 |
| 233 | 2689 | 0.494 | 334.3 | 24 | 3.3 | 23.8 | 1.2 | 2 | -4.044 | -5.73 | -7.821 | -0.168 |
| 234 | 24964624 | 0.457 | 407.5 | 31 | 4.4 | 83.6 | 1 | 4 | -8.022 | -10.419 | -7.82 | -0.259 |
| 235 | 9966051 | 0.388 | 298.4 | 21 | 3.9 | 15.3 | 3.2 | 3 | -6.135 | -6.998 | -7.802 | -0.292 |
| 236 | 44468216 | 0.5 | 291.3 | 21 | -0.7 | 149.9 | 5.5 | 5 | -5.759 | -7.614 | -7.78 | -0.274 |
| 237 | 5281107 | 0.316 | 412.7 | 30 | 7 | 40.5 | 8.5 | 7 | -9.567 | -9.352 | -7.76 | -0.319 |
| 238 | 10093303 | 0.391 | 415.5 | 29 | -0.3 | 162.6 | 13 | 14 | -9.088 | -9.952 | -7.701 | -0.313 |
| 239 | 135398748 | 0.5 | 253.3 | 18 | -0.7 | 125.8 | 7.2 | 7 | -5.329 | -6.94 | -7.689 | -0.296 |
| 240 | 9889366 | 0.525 | 438.6 | 29 | 2.9 | 92.3 | 8 | 10 | -7.3 | -8.448 | -7.687 | -0.252 |
| 241 | 10071166 | 0.371 | 427.4 | 28 | 6.4 | 63.6 | 8.4 | 10 | -7.906 | -8.708 | -7.667 | -0.282 |
| 242 | 114745 | 0.25 | 300.5 | 21 | 5.1 | 15.6 | 2.3 | 3 | -6.631 | -7.414 | -7.667 | -0.316 |
| 243 | 57267 | 0.75 | 340.8 | 23 | 2.9 | 61.8 | 5.8 | 7 | -7.878 | -7.894 | -7.66 | -0.343 |
| 244 | 46926973 | 0.488 | 396.5 | 30 | 3.8 | 93.5 | 2.3 | 5 | -7.65 | -9.676 | -7.659 | -0.255 |
| 245 | 17134 | 0.522 | 310.3 | 21 | 0.9 | 116.4 | 2.8 | 5 | -6.324 | -7.246 | -7.628 | -0.301 |
| 246 | 2726 | 0.262 | 318.9 | 21 | 4.9 | 6.5 | 4 | 4 | -6.412 | -7.012 | -7.592 | -0.305 |
| 247 | 37464 | 0.567 | 291.4 | 21 | 3.9 | 41.5 | 6 | 8 | -8.937 | -9.296 | -7.583 | -0.426 |
| 248 | 72136 | 0.223 | 366.4 | 25 | 5.5 | 6.5 | 4 | 5 | -5.8 | -7.094 | -7.582 | -0.232 |
| 249 | 447715 | 0.382 | 420.6 | 30 | 5.9 | 77.8 | 10.1 | 8 | -9.29 | -9.568 | -7.579 | -0.31 |
| 250 | 447043 | 0.15 | 749 | 52 | 3.8 | 180.1 | 21.4 | 12 | -7.987 | -11.395 | -7.578 | -0.154 |
| 251 | 3290 | 0.25 | 312.5 | 22 | 5 | 6.5 | 5 | 5 | -5.901 | -6.664 | -7.557 | -0.268 |
| 252 | 10206 | 0.25 | 606.7 | 45 | 6.9 | 61.9 | 3.2 | 2 | -9.657 | -11.34 | -7.549 | -0.215 |
| 253 | 9956222 | 0.304 | 467.5 | 35 | 2.9 | 132.3 | 1.7 | 4 | -8.386 | -10.963 | -7.505 | -0.24 |
| 254 | 2726 | 0.262 | 318.9 | 21 | 4.9 | 6.5 | 4 | 4 | -6.461 | -7.117 | -7.494 | -0.308 |
| 255 | 25147749 | 0.322 | 406.6 | 27 | 5.3 | 40 | 7.4 | 6 | -8.57 | -9.255 | -7.438 | -0.317 |
| 256 | 56649450 | 0.365 | 441.5 | 30 | 3.8 | 101.2 | 3.3 | 7 | -6.969 | -8.817 | -7.433 | -0.232 |
| 257 | 11582982 | 0.477 | 438.6 | 30 | 1.9 | 100.1 | 8.3 | 6 | -10.088 | -10.676 | -7.427 | -0.336 |
| 258 | 253602 | 0.75 | 265.3 | 19 | 0.9 | 67.8 | 5 | 5 | -7.243 | -7.799 | -7.406 | -0.381 |
| 259 | 5280723 | 0.513 | 354.5 | 25 | 4.1 | 94.8 | 15.8 | 16 | -7.786 | -9.31 | -7.405 | -0.311 |
| 260 | 2475 | 0.654 | 271.8 | 18 | 3.2 | 41.5 | 5.5 | 7 | -8.157 | -8.716 | -7.379 | -0.453 |
| 261 | 5287705 | 0.394 | 340.5 | 25 | 6 | 37.3 | 1.3 | 7 | -7.013 | -8.145 | -7.335 | -0.281 |
| 262 | 9924495 | 0.588 | 349.4 | 27 | 4.3 | 57 | 1.4 | 3 | -5.455 | -8.555 | -7.283 | -0.202 |
| 263 | 24776445 | 0.406 | 421.3 | 27 | 4.8 | 76.1 | 1.5 | 4 | -8.017 | -10.373 | -7.262 | -0.297 |
| 264 | 70945511 | 0.25 | 535.6 | 36 | 2.2 | 121.4 | 12.3 | 14 | -7.764 | -9.425 | -7.244 | -0.216 |
| 265 | 2724387 | 0.5 | 299.3 | 20 | -0.9 | 138.1 | 4.6 | 5 | -4.578 | -7.395 | -7.208 | -0.229 |
| 266 | 6197 | 0.728 | 281.4 | 20 | 1.4 | 83.5 | 5.9 | 4 | -7.083 | -7.958 | -7.184 | -0.354 |
| 267 | 6238 | 0.613 | 330.5 | 24 | 4.1 | 54.4 | 4.3 | 2 | -7.278 | -7.386 | -7.182 | -0.303 |
| 268 | 448537 | 0.446 | 268.4 | 20 | 4.8 | 40.5 | 3.5 | 6 | -5.846 | -6.287 | -7.174 | -0.292 |
| 269 | 570342 | 0.662 | 327.3 | 25 | 3.7 | 63.2 | 0.5 | 2 | -6.122 | -8.05 | -7.145 | -0.245 |
| 270 | 32798 | 0.388 | 467 | 32 | 4.3 | 80.7 | 6.8 | 5 | -5.614 | -8.215 | -7.085 | -0.175 |
| 271 | 5284513 | 0.496 | 326.4 | 24 | 4.8 | 46.5 | 2 | 7 | -6.312 | -7.131 | -6.992 | -0.263 |
| 272 | 2794 | 0.211 | 473.4 | 33 | 7.6 | 40 | 2 | 4 | -7.391 | -9.078 | -6.942 | -0.224 |
| 273 | 53389 | 0.389 | 321.5 | 24 | 3.9 | 20.2 | 5 | 4 | -8.04 | -10.019 | -6.933 | -0.335 |
| 274 | 10173277 | 0.593 | 418.5 | 31 | 3.1 | 84.8 | 5.7 | 8 | -7.924 | -11.127 | -6.915 | -0.256 |
| 275 | 3603 | 0.279 | 349.6 | 24 | 5.3 | 23.5 | 8.9 | 7 | -7.939 | -5.959 | -6.894 | -0.331 |
| 276 | 9444 | 0.5 | 244.2 | 17 | -1.7 | 141 | 4.7 | 5 | -3.509 | -6.16 | -6.88 | -0.206 |
| 277 | 37542 | 0.5 | 244.2 | 17 | -1.9 | 143.7 | 4.8 | 6 | -5.665 | -8.276 | -6.868 | -0.333 |
| 278 | 10141893 | 0.25 | 503.3 | 29 | 5 | 71.1 | 3.7 | 5 | -7.616 | -9.962 | -6.83 | -0.263 |
| 279 | 131664 | 0.404 | 407.9 | 29 | 5.4 | 63.2 | 5.3 | 6 | -7.38 | -9.544 | -6.829 | -0.254 |
| 280 | 2540 | 0 | 610.7 | 45 | 6.3 | 143.3 | 8.2 | 12 | -9.302 | -12.05 | -6.748 | -0.207 |
| 281 | 51634 | 0.724 | 219.3 | 15 | 0.1 | 84.2 | 9 | 8 | -6.22 | -8.02 | -6.72 | -0.415 |
| 282 | 10323598 | 0.293 | 497.6 | 37 | 4.5 | 83.7 | 2.2 | 6 | -8.223 | -11.145 | -6.708 | -0.222 |
| 283 | 151171 | 0.252 | 498.6 | 38 | 6.5 | 78.1 | 1.6 | 5 | -7.17 | -8.758 | -6.688 | -0.189 |
| 284 | 501640 | 0.724 | 289.4 | 20 | 2 | 84.2 | 14 | 13 | -10.114 | -10.396 | -6.686 | -0.506 |
| 285 | 151194 | 0.5 | 346.8 | 25 | 5 | 50.7 | 2.5 | 4 | -6.912 | -8.791 | -6.685 | -0.276 |
| 286 | 1894361 | 0.213 | 392.5 | 30 | 5.7 | 24.1 | 9 | 9 | -7.857 | -10.684 | -6.684 | -0.262 |
| 287 | 73078 | 0.25 | 622.8 | 46 | 7.2 | 61.9 | 4 | 4 | -7.487 | -9.839 | -6.657 | -0.163 |
| 288 | 441383 | 0.313 | 532.5 | 36 | 4.5 | 64.9 | 7.2 | 8 | -9.637 | -12.416 | -6.581 | -0.268 |
| 289 | 8814 | 0.364 | 206.3 | 15 | 4.1 | 20.2 | 2.5 | 4 | -4.703 | -6.077 | -6.579 | -0.314 |
| 290 | 60750 | 0.573 | 263.2 | 18 | -0.4 | 108.4 | 3.7 | 4 | -3.957 | -6.403 | -6.523 | -0.22 |
| 291 | 4034 | 0.182 | 391 | 28 | 5.6 | 6.5 | 6 | 5 | -7.604 | -9.004 | -6.44 | -0.272 |
| 292 | 7098680 | 0.25 | 606.7 | 45 | 6.9 | 61.9 | 3.2 | 2 | -8.328 | -10.349 | -6.436 | -0.185 |
| 293 | 24800108 | 0.261 | 408.6 | 30 | 5.6 | 33.1 | 5.9 | 3 | -8.538 | -11.754 | -6.365 | -0.285 |
| 294 | 59472121 | 0.261 | 463.6 | 33 | 3.4 | 124 | 4 | 7 | -6.986 | -9.951 | -6.333 | -0.212 |
| 295 | 46216796 | 0.321 | 472.6 | 35 | 4.8 | 68.7 | 6 | 4 | -8.53 | -10.641 | -6.33 | -0.244 |
| 296 | 25150857 | 0.403 | 490.6 | 36 | 3.9 | 66 | 7.6 | 7 | -9.494 | -11.75 | -6.309 | -0.264 |
| 297 | 5311382 | 0.25 | 512.4 | 35 | 5.8 | 70.6 | 6.2 | 9 | -8.114 | -10.885 | -6.283 | -0.232 |
| 298 | 121926 | 0.483 | 390.4 | 29 | 4.6 | 73.4 | 5.8 | 7 | -6.742 | -8.907 | -6.275 | -0.232 |
| 299 | 5328940 | 0.232 | 530.5 | 36 | 5.2 | 82.9 | 8 | 9 | -9.231 | -11.683 | -6.27 | -0.256 |
| 300 | 108144 | 0.357 | 424.7 | 27 | 3.3 | 116.9 | 2.8 | 5 | -8.502 | -10.684 | -6.27 | -0.315 |
| 301 | 119031 | 0.5 | 331.4 | 23 | 0.2 | 127.8 | 9 | 10 | -6.049 | -7.696 | -6.235 | -0.263 |
| 302 | 2782 | 0.334 | 325.8 | 23 | 4.4 | 21.1 | 4.5 | 4 | -7.574 | -9.704 | -6.233 | -0.329 |
| 303 | 25260757 | 0.214 | 519.6 | 38 | 3.5 | 115.7 | 3.6 | 6 | -8.642 | -10.551 | -6.224 | -0.227 |
| 304 | 2723601 | 0.75 | 167.2 | 11 | -0.2 | 79.1 | 0 | 0 | -5.736 | -6.5 | -6.218 | -0.521 |
| 305 | 10219 | 0.295 | 480.6 | 35 | 4.9 | 52.2 | 6.8 | 7 | -9.096 | -10.591 | -6.217 | -0.26 |
| 306 | 3033832 | 0.5 | 192.1 | 13 | -0.9 | 132.1 | 6.8 | 9 | -5.667 | -5.999 | -6.207 | -0.436 |
| 307 | 6729 | 0.112 | 433 | 31 | 6.6 | 6.5 | 6 | 6 | -7.914 | -9.263 | -6.154 | -0.255 |
| 308 | 4030 | 0.724 | 295.3 | 22 | 3 | 84.1 | 1.3 | 5 | -5.312 | -7.253 | -6.101 | -0.241 |
| 309 | 68617 | 0.25 | 306.2 | 20 | 5.2 | 12 | 1.5 | 2 | -7.825 | -9.163 | -6.026 | -0.391 |
| 310 | 2733525 | 0.5 | 192.1 | 13 | -0.9 | 132.1 | 6.8 | 9 | -5.64 | -6.244 | -6.018 | -0.434 |
| 311 | 10182969 | 0.375 | 459.5 | 34 | 2.7 | 110.8 | 2.9 | 5 | -7.583 | -9.502 | -6.002 | -0.223 |
| 312 | 50905713 | 0.409 | 416.9 | 30 | 4.5 | 86.8 | 2.7 | 4 | -6.956 | -9.132 | -5.999 | -0.232 |
| 313 | 4680 | 0.636 | 339.4 | 25 | 3.9 | 49.8 | 4 | 6 | -7.069 | -8.483 | -5.995 | -0.283 |
| 314 | 11313622 | 0.422 | 334.4 | 24 | 5.1 | 40.6 | 2.8 | 3 | -5.436 | -6.888 | -5.993 | -0.227 |
| 315 | 22227931 | 0.297 | 505 | 36 | 4.3 | 86.4 | 4.1 | 5 | -7.027 | -9.201 | -5.991 | -0.195 |
| 316 | 3336 | 0.25 | 315.5 | 24 | 5.6 | 12 | 7 | 7 | -5.742 | -7.518 | -5.974 | -0.239 |
| 317 | 130787 | 0.338 | 329.4 | 25 | 4.3 | 18.8 | 1.2 | 2 | -6.426 | -7.904 | -5.963 | -0.257 |
| 318 | 148195 | 0.25 | 638.8 | 36 | 6.1 | 79.5 | 4.7 | 5 | -9.546 | -10.359 | -5.953 | -0.265 |
| 319 | 3005572 | 0.5 | 192.1 | 13 | -0.9 | 132.1 | 6.8 | 9 | -5.799 | -6.057 | -5.951 | -0.446 |
| 320 | 2577 | 0.75 | 257.3 | 18 | 1.6 | 78.5 | 6 | 7 | -4.903 | -6.492 | -5.931 | -0.272 |
| 321 | 54684141 | 0.75 | 270.2 | 19 | 3 | 73.1 | 2 | 4 | -3.739 | -5.513 | -5.887 | -0.197 |
| 322 | 2738575 | 0.75 | 300.4 | 22 | 0.8 | 61.6 | 3.5 | 4 | -6.018 | -8.345 | -5.885 | -0.274 |
| 323 | 2880 | 0.358 | 310.8 | 22 | 4.6 | 27 | 3 | 5 | -6.629 | -7.678 | -5.879 | -0.301 |
| 324 | 9845807 | 0.216 | 434.6 | 32 | 5.3 | 32.8 | 8.7 | 8 | -8.352 | -9.71 | -5.87 | -0.261 |
| 325 | 8395 | 0.725 | 324.3 | 24 | 3.2 | 61.8 | 3.5 | 5 | -6.131 | -7.785 | -5.858 | -0.255 |
| 326 | 65776 | 0.495 | 350.5 | 26 | 4 | 34.5 | 5.3 | 6 | -7.457 | -9.901 | -5.831 | -0.287 |
| 327 | 219104 | 0.573 | 188.3 | 11 | 2.9 | 28.7 | 4 | 4 | -6.874 | -7.802 | -5.815 | -0.625 |
| 328 | 3036780 | 0.338 | 285.8 | 20 | 4.3 | 12.5 | 0.5 | 0 | -6.562 | -7.489 | -5.815 | -0.328 |
| 329 | 26987 | 0.25 | 343.9 | 24 | 5.1 | 12.5 | 6.5 | 6 | -7.939 | -9.665 | -5.806 | -0.331 |
| 330 | 214347 | 0.525 | 329.4 | 25 | 4.8 | 73.6 | 2 | 4 | -6.237 | -7.438 | -5.798 | -0.249 |
| 331 | 2800 | 0.157 | 406 | 29 | 6.6 | 12.5 | 6.3 | 9 | -7.17 | -9.833 | -5.792 | -0.247 |
| 332 | 10219 | 0.295 | 480.6 | 35 | 4.9 | 52.2 | 6.8 | 7 | -9.533 | -11.625 | -5.788 | -0.272 |
| 333 | 4921 | 0 | 844.4 | 60 | 6.2 | 139.7 | 20.5 | 23 | -12.405 | -15.762 | -5.749 | -0.207 |
| 334 | 3957 | 0.394 | 382.9 | 27 | 4.9 | 42.4 | 2.4 | 3 | -7.453 | -8.332 | -5.745 | -0.276 |
| 335 | 2733525 | 0.214 | 371.5 | 28 | 6 | 12.5 | 5.3 | 8 | -7.684 | -10.202 | -5.727 | -0.274 |
| 336 | 163091 | 0.338 | 285.8 | 20 | 4.3 | 12.5 | 0.5 | 0 | -6.753 | -7.63 | -5.718 | -0.338 |
| 337 | 67683363 | 0 | 883 | 65 | 7.7 | 193.1 | 10.7 | 17 | -10.793 | -12.798 | -5.717 | -0.166 |
| 338 | 4011 | 0.35 | 277.4 | 21 | 4.2 | 12 | 4.5 | 4 | -7.169 | -8.817 | -5.713 | -0.341 |
| 339 | 44247568 | 0.662 | 337.4 | 25 | 3 | 39.5 | 4.7 | 5 | -5.806 | -8.44 | -5.71 | -0.232 |
| 340 | 71661251 | 0 | 882 | 65 | 8.1 | 188.8 | 8.8 | 17 | -9.459 | -12.139 | -5.706 | -0.146 |
| 341 | 444795 | 0.394 | 300.4 | 22 | 5.2 | 37.3 | 2 | 6 | -6.419 | -7.64 | -5.697 | -0.292 |
| 342 | 4594 | 0.75 | 345.4 | 24 | 2.9 | 77.1 | 4 | 5 | -6.774 | -9.172 | -5.677 | -0.282 |
| 343 | 49803313 | 0.25 | 552.7 | 40 | 2.7 | 121.1 | 6.8 | 9 | -10.115 | -11.925 | -5.675 | -0.253 |
| 344 | 23631927 | 0.213 | 535.6 | 39 | 3.3 | 141.9 | 8.7 | 13 | -7.589 | -8.865 | -5.649 | -0.195 |
| 345 | 9821849 | 0.341 | 391.6 | 29 | 5.8 | 39.2 | 2.8 | 2 | -7.955 | -9.49 | -5.635 | -0.274 |
| 346 | 5719 | 0.75 | 305.3 | 23 | 2.6 | 74.3 | 2.5 | 4 | -5.291 | -6.934 | -5.612 | -0.23 |
| 347 | 135413553 | 0.438 | 300.8 | 21 | 4.4 | 33.6 | 1.7 | 2 | -6.387 | -8.254 | -5.607 | -0.304 |
| 348 | 2733526 | 0.214 | 371.5 | 28 | 6 | 12.5 | 5.3 | 8 | -7.452 | -10.034 | -5.599 | -0.266 |
| 349 | 3005572 | 0.157 | 406 | 29 | 6.2 | 12.5 | 6.3 | 9 | -8.054 | -10.364 | -5.571 | -0.278 |
| 350 | 456201 | 0.338 | 531.4 | 36 | 4.3 | 69.1 | 6.5 | 8 | -9.089 | -11.743 | -5.567 | -0.252 |
| 351 | 3033832 | 0.157 | 406 | 29 | 6.6 | 12.5 | 6.3 | 9 | -7.38 | -9.887 | -5.566 | -0.254 |
| 352 | 24868317 | 0.29 | 350.5 | 27 | 5.2 | 24.9 | 6 | 6 | -6.335 | -8.593 | -5.532 | -0.235 |
| 353 | 3456 | 0.142 | 414.6 | 31 | 5 | 15.7 | 11 | 10 | -7.708 | -10.195 | -5.531 | -0.249 |
| 354 | 3005573 | 0.157 | 406 | 29 | 6.2 | 12.5 | 6.3 | 9 | -8.223 | -10.738 | -5.528 | -0.284 |
| 355 | 153711 | 0.511 | 364.4 | 24 | 0 | 114.4 | 7 | 6 | -3.451 | -6.821 | -5.457 | -0.144 |
| 356 | 2267 | 0.417 | 381.9 | 27 | 4.3 | 35.9 | 4.3 | 3 | -7.298 | -9.073 | -5.455 | -0.27 |
| 357 | 2344 | 0.325 | 307.4 | 23 | 4.4 | 12.5 | 5.1 | 4 | -6.737 | -8.101 | -5.452 | -0.293 |
| 358 | 5353853 | 0.28 | 429.1 | 26 | 6.2 | 39.4 | 5 | 6 | -8.418 | -10.296 | -5.442 | -0.324 |
| 359 | 1102 | 0.75 | 145.2 | 10 | -0.3 | 64.1 | 9 | 9 | -7.139 | -10.135 | -5.441 | -0.714 |
| 360 | 71667668 | 0.25 | 562.7 | 41 | 2.1 | 120.2 | 6.6 | 10 | -10.068 | -11.233 | -5.413 | -0.246 |
| 361 | 126388 | 0.425 | 301.3 | 19 | 3.6 | 6.5 | 6.5 | 6 | -8.184 | -10.661 | -5.411 | -0.431 |
| 362 | 2949965 | 0.5 | 318.4 | 24 | 4.4 | 41 | 3 | 4 | -6.519 | -7.714 | -5.383 | -0.272 |
| 363 | 71821 | 0.363 | 256.7 | 18 | 4.1 | 17.8 | 2 | 2 | -5.931 | -7.824 | -5.362 | -0.329 |
| 364 | 57379345 | 0.092 | 558.1 | 38 | 6.4 | 105.2 | 5.5 | 9 | -8.841 | -10.013 | -5.336 | -0.233 |
| 365 | 2805 | 0.25 | 329.9 | 23 | 5 | 12.5 | 7 | 6 | -7.763 | -9.606 | -5.324 | -0.338 |
| 366 | 7547 | 0.426 | 315.6 | 19 | 5.4 | 41.1 | 1 | 4 | -5.033 | -6.602 | -5.261 | -0.265 |
| 367 | 492405 | 0.721 | 157.1 | 11 | -1.3 | 84.6 | 0.4 | 1 | -3.159 | -4.675 | -5.261 | -0.287 |
| 368 | 53315868 | 0.25 | 635.9 | 46 | 6.3 | 69.2 | 14.5 | 12 | -11.556 | -14.015 | -5.22 | -0.251 |
| 369 | 10223146 | 0.385 | 408.3 | 27 | 4.3 | 96.8 | 3 | 6 | -7.71 | -8.794 | -5.212 | -0.286 |
| 370 | 5353365 | 0.75 | 330.8 | 23 | 2.9 | 58.4 | 1 | 2 | -6.772 | -7.633 | -5.202 | -0.294 |
| 371 | 2051 | 0.613 | 315.8 | 22 | 4.1 | 56.3 | 1.5 | 4 | -5.632 | -7.376 | -5.198 | -0.256 |
| 372 | 3280 | 0.422 | 395.5 | 29 | 5.4 | 49.8 | 8 | 10 | -7.078 | -8.953 | -5.196 | -0.244 |
| 373 | 3198 | 0.256 | 381.7 | 24 | 5.9 | 27 | 6 | 6 | -7.881 | -9.736 | -5.192 | -0.328 |
| 374 | 1201549 | 0.325 | 307.4 | 23 | 4.4 | 12.5 | 5.1 | 4 | -6.522 | -7.943 | -5.16 | -0.284 |
| 375 | 3038495 | 0.444 | 417.5 | 31 | 4.2 | 44.8 | 7 | 8 | -6.138 | -8.099 | -5.144 | -0.198 |
| 376 | 671690 | 0.479 | 314.2 | 20 | 3.8 | 29.5 | 2.4 | 2 | -6.626 | -7.338 | -5.135 | -0.331 |
| 377 | 178144 | 0.647 | 331.5 | 24 | 2.5 | 37.7 | 6.3 | 7 | -7.256 | -9.09 | -5.128 | -0.302 |
| 378 | 29393 | 0.5 | 346.4 | 23 | 1.6 | 155 | 6.8 | 9 | -4.27 | -6.701 | -5.125 | -0.186 |
| 379 | 11755771 | 0.29 | 502 | 35 | 3 | 113.6 | 13.5 | 15 | -8.259 | -9.911 | -5.124 | -0.236 |
| 380 | 9801 | 0.25 | 329.5 | 25 | 5.4 | 12 | 8 | 8 | -5.036 | -7.093 | -5.094 | -0.201 |
| 381 | 4008 | 0.044 | 610.7 | 45 | 4.8 | 116.9 | 7.7 | 10 | -9.158 | -11.577 | -5.089 | -0.204 |
| 382 | 10201696 | 0.594 | 411.5 | 30 | 2.3 | 88.5 | 8.4 | 7 | -8.279 | -11.25 | -5.058 | -0.276 |
| 383 | 1712095 | 0.424 | 397.5 | 29 | 4.9 | 48.9 | 6.7 | 9 | -7.698 | -9.304 | -5.037 | -0.265 |
| 384 | 72430 | 0.668 | 382.5 | 28 | 2.9 | 84.5 | 9.5 | 11 | -6.874 | -8.362 | -5.033 | -0.245 |
| 385 | 4239764 | 0.537 | 395.3 | 21 | 4.1 | 50.4 | 3.7 | 3 | -6.333 | -8.084 | -5.031 | -0.302 |
| 386 | 65866 | 0.039 | 611.7 | 45 | 6.4 | 113.7 | 8.8 | 12 | -7.463 | -9.37 | -4.987 | -0.166 |
| 387 | 68873 | 0.517 | 366.5 | 27 | 4.2 | 43.4 | 3.7 | 0 | -7.211 | -8.167 | -4.972 | -0.267 |
| 388 | 3191 | 0.13 | 469.7 | 35 | 7.2 | 29.5 | 9.2 | 10 | -7.957 | -9.507 | -4.965 | -0.227 |
| 389 | 3103 | 0.388 | 281.4 | 21 | 3.9 | 12.5 | 5 | 4 | -6.042 | -7.445 | -4.954 | -0.288 |
| 390 | 71496458 | 0.279 | 499.6 | 37 | 4.4 | 87.5 | 4.8 | 10 | -7.443 | -10.102 | -4.95 | -0.201 |
| 391 | 5375570 | 0.75 | 199.3 | 12 | 1.3 | 50.4 | 1 | 3 | -5.589 | -6.275 | -4.92 | -0.466 |
| 392 | 115210 | 0.625 | 312.4 | 23 | 4 | 64.2 | 2.8 | 4 | -6.197 | -7.267 | -4.897 | -0.269 |
| 393 | 40326 | 0.31 | 391.3 | 26 | 6 | 35.5 | 5 | 6 | -7.958 | -9.378 | -4.883 | -0.306 |
| 394 | 439731 | 0.687 | 150.1 | 10 | -1.1 | 90.1 | 5 | 4 | -3.468 | -6.196 | -4.867 | -0.347 |
| 395 | 680502 | 0.568 | 337.4 | 25 | 4.3 | 47.6 | 1.2 | 4 | -5.686 | -7.11 | -4.836 | -0.227 |
| 396 | 4413 | 0.5 | 347.4 | 26 | 2.2 | 140.6 | 1.5 | 4 | -5.598 | -7.184 | -4.829 | -0.215 |
| 397 | 4497 | 0.388 | 418.4 | 30 | 2.9 | 119.7 | 4.8 | 8 | -6.112 | -8.05 | -4.828 | -0.204 |
| 398 | 5770 | 0.114 | 608.7 | 44 | 4.2 | 117.8 | 7.3 | 8 | -9.027 | -10.775 | -4.811 | -0.205 |
| 399 | 4055 | 0.618 | 172.2 | 13 | 1.9 | 34.1 | 0 | 0 | -3.877 | -4.863 | -4.784 | -0.298 |
| 400 | 2162 | 0.527 | 408.9 | 28 | 2.3 | 99.9 | 6.5 | 9 | -7.845 | -9.849 | -4.758 | -0.28 |
| 401 | 44093 | 0.75 | 217.3 | 14 | 0.6 | 57.6 | 5 | 6 | -5.131 | -6.012 | -4.737 | -0.366 |
| 402 | 237 | 0.312 | 400 | 28 | 6 | 37.4 | 7.8 | 9 | -7.822 | -9.624 | -4.731 | -0.279 |
| 403 | 6167 | 0.648 | 399.4 | 29 | 2.4 | 83.1 | 3.6 | 5 | -6.198 | -6.996 | -4.731 | -0.214 |
| 404 | 2442 | 0.321 | 376.1 | 18 | 4.7 | 29.3 | 4 | 3 | -7.857 | -8.051 | -4.694 | -0.436 |
| 405 | 2801 | 0.3 | 314.9 | 22 | 4.6 | 6.5 | 4.3 | 4 | -6.608 | -7.112 | -4.683 | -0.3 |
| 406 | 2801 | 0.3 | 314.9 | 22 | 4.6 | 6.5 | 4.3 | 4 | -6.857 | -8.699 | -4.668 | -0.312 |
| 407 | 177992 | 0.196 | 564.7 | 41 | 4.4 | 100.6 | 9.5 | 12 | -8.444 | -9.454 | -4.635 | -0.206 |
| 408 | 2157 | 0.189 | 645.3 | 31 | 6.9 | 42.7 | 9 | 11 | -8.761 | -9.818 | -4.6 | -0.283 |
| 409 | 2536 | 0.419 | 398.4 | 29 | 1 | 137.3 | 5.5 | 8 | -6.481 | -8.43 | -4.538 | -0.223 |
| 410 | 25382 | 0.3 | 291.4 | 22 | 4.6 | 3.2 | 3 | 3 | -6.406 | -8.256 | -4.532 | -0.291 |
| 411 | 5475 | 0.42 | 410.6 | 30 | 4.3 | 42 | 11 | 12 | -6.635 | -9.641 | -4.518 | -0.221 |
| 412 | 44224135 | 0.54 | 401.9 | 28 | 2.4 | 99.8 | 6.3 | 7 | -6.961 | -7.99 | -4.475 | -0.249 |
| 413 | 21330 | 0.75 | 267.3 | 18 | 1.3 | 72.5 | 1 | 2 | -5.36 | -6.876 | -4.471 | -0.298 |
| 414 | 71081 | 0.748 | 88 | 6 | -0.8 | 80.3 | 0.3 | 1 | -3.388 | -4.186 | -4.437 | -0.565 |
| 415 | 443955 | 0.483 | 350.5 | 26 | 4.1 | 34.5 | 3.5 | 3 | -6.198 | -6.529 | -4.413 | -0.238 |
| 416 | 3194 | 0.503 | 274.2 | 16 | 2.4 | 20.3 | 0.3 | 1 | -3.772 | -5.396 | -4.326 | -0.236 |
| 417 | 13919623 | 0.695 | 246.3 | 18 | 2.4 | 43.4 | 1 | 0 | -4.89 | -6.128 | -4.324 | -0.272 |
| 418 | 3025 | 0.688 | 343.5 | 25 | 3.5 | 54.5 | 8.8 | 10 | -7.585 | -9.575 | -4.309 | -0.303 |
| 419 | 3805581 | 0.431 | 291.8 | 20 | 4.1 | 28.2 | 6.3 | 7 | -7.053 | -8.496 | -4.294 | -0.353 |
| 420 | 17970 | 0.429 | 381.5 | 28 | 4.4 | 38.8 | 9 | 8 | -6.715 | -8.261 | -4.287 | -0.24 |
| 421 | 5475 | 0.42 | 410.6 | 30 | 4.3 | 42 | 11 | 12 | -7.854 | -10.057 | -4.261 | -0.262 |
| 422 | 2719 | 0.343 | 319.9 | 22 | 4.8 | 28.2 | 7.3 | 8 | -7.502 | -9.168 | -4.191 | -0.341 |
| 423 | 4124851 | 0.672 | 222.3 | 15 | 2.3 | 40.6 | 2.5 | 2 | -3.602 | -4.69 | -4.186 | -0.24 |
| 424 | 4746 | 0.25 | 277.5 | 20 | 5.3 | 12 | 6.9 | 4 | -7.015 | -8.529 | -4.176 | -0.351 |
| 425 | 4756 | 0.689 | 213.2 | 16 | 2.7 | 89.7 | 0.8 | 2 | -4.645 | -5.626 | -4.173 | -0.29 |
| 426 | 2520 | 0.326 | 454.6 | 33 | 5.1 | 64 | 12 | 13 | -7.073 | -8.853 | -3.994 | -0.214 |
| 427 | 462382 | 0.256 | 475.6 | 34 | 3.6 | 113.6 | 14.5 | 16 | -8.172 | -9.586 | -3.958 | -0.24 |
| 428 | 6051 | 0.662 | 197.2 | 15 | 3.7 | 50.7 | 0.8 | 2 | -3.014 | -4.301 | -3.923 | -0.201 |
| 429 | 169870 | 0.369 | 428.6 | 31 | 5.9 | 60.4 | 8.3 | 6 | -7.503 | -8.664 | -3.869 | -0.242 |
| 430 | 4168 | 0.75 | 299.8 | 20 | 2 | 67.6 | 5.8 | 7 | -6.844 | -8.323 | -3.825 | -0.342 |
| 431 | 2719 | 0.343 | 319.9 | 22 | 4.8 | 28.2 | 7.3 | 8 | -7.236 | -7.914 | -3.8 | -0.329 |
| 432 | 154573793 | 0.341 | 445.5 | 32 | 1.5 | 142.6 | 12.3 | 15 | -6.481 | -9.135 | -3.781 | -0.203 |
| 433 | 2754 | 0.643 | 369.5 | 27 | 3.5 | 81.9 | 6.9 | 7 | -6.45 | -7.265 | -3.722 | -0.239 |
| 434 | 160512 | 0.375 | 216.3 | 16 | 4 | 17.1 | 3.3 | 4 | -5.559 | -6.473 | -3.685 | -0.347 |
| 435 | 6476696 | 0.744 | 353.6 | 25 | 1.4 | 60.1 | 20 | 20 | -9.833 | -12.408 | -3.472 | -0.393 |
| 436 | 14632996 | 0.4 | 218.3 | 16 | 3.8 | 17.1 | 3.4 | 4 | -5.155 | -6.147 | -3.415 | -0.322 |
| 437 | 10391 | 0.576 | 197.7 | 13 | 1.9 | 29.1 | 4 | 4 | -4.538 | -5.215 | -3.314 | -0.349 |
| 438 | 10324367 | 0.25 | 519.7 | 37 | 1.7 | 150.7 | 8.8 | 13 | -7.857 | -8.966 | -3.201 | -0.212 |
| 439 | 4499 | 0.484 | 388.4 | 28 | 3.1 | 110.4 | 2.8 | 6 | -4.765 | -6.047 | -3.144 | -0.17 |
| 440 | 73364 | 0.676 | 362.5 | 26 | 3.2 | 84.5 | 11.5 | 13 | -6.187 | -7.61 | -3.083 | -0.238 |
| 441 | 196216 | 0.4 | 218.3 | 16 | 3.8 | 17.1 | 3.7 | 4 | -5.634 | -6.294 | -2.891 | -0.352 |
| 442 | 3117 | 0.425 | 296.5 | 16 | 3.6 | 6.5 | 7.5 | 9 | -7.046 | -7.87 | -2.848 | -0.44 |
| 443 | 443118 | 0.542 | 383.5 | 27 | 1.9 | 104.4 | 13 | 13 | -6.681 | -8.365 | -2.772 | -0.247 |
| 444 | 4485 | 0.56 | 346.3 | 25 | 2.1 | 110.4 | 0.8 | 4 | -4.844 | -5.838 | -2.74 | -0.194 |
| 445 | 65663 | 0.644 | 342.4 | 24 | 1 | 97 | 10 | 10 | -6.561 | -7.267 | -2.585 | -0.273 |
| 446 | 2683 | 0.25 | 304.5 | 22 | 7.3 | 3.9 | 15 | 15 | -6.359 | -7.304 | -2.502 | -0.289 |
| 447 | 9952916 | 0.396 | 401.6 | 29 | 7.7 | 47.9 | 15.7 | 16 | -7.273 | -8.089 | -2.261 | -0.251 |
| 448 | 2733491 | 0.372 | 426.6 | 30 | -0.2 | 168.8 | 14.3 | 14 | -7.643 | -9.67 | -2.227 | -0.255 |
| 449 | 51167 | 0.093 | 550.9 | 40 | 9.8 | 31.2 | 25.5 | 25 | -8.429 | -10.989 | -0.972 | -0.211 |
| 450 | 4581100 | 0.089 | 477.8 | 33 | 9.5 | 26.3 | 21.3 | 22 | -7.052 | -7.637 | 0.02 | -0.214 |
